# Physiologically Based Pharmacokinetic Modeling of Transporter-Mediated Hepatic Disposition of Imaging Biomarker Gadoxetate in Rats

**DOI:** 10.1021/acs.molpharmaceut.1c00206

**Published:** 2021-07-20

**Authors:** Daniel Scotcher, Nicola Melillo, Sirisha Tadimalla, Adam S. Darwich, Sabina Ziemian, Kayode Ogungbenro, Gunnar Schütz, Steven Sourbron, Aleksandra Galetin

**Affiliations:** † Centre for Applied Pharmacokinetic Research, School of Health Sciences, University of Manchester, Manchester M13 9PL, U.K.; ‡ Division of Medical Physics, University of Leeds, Leeds LS2 9JT, U.K.; § MR & CT Contrast Media Research, Bayer AG, Berlin 13342, Germany; ∥ Department of Infection, Immunity and Cardiovascular Disease, University of Sheffield, Sheffield S10 2TN, U.K.

**Keywords:** gadoxetate, imaging biomarker, drug transporters, physiologically based pharmacokinetic model, hepatobiliary excretion, drug−drug interactions, quantitative translation

## Abstract

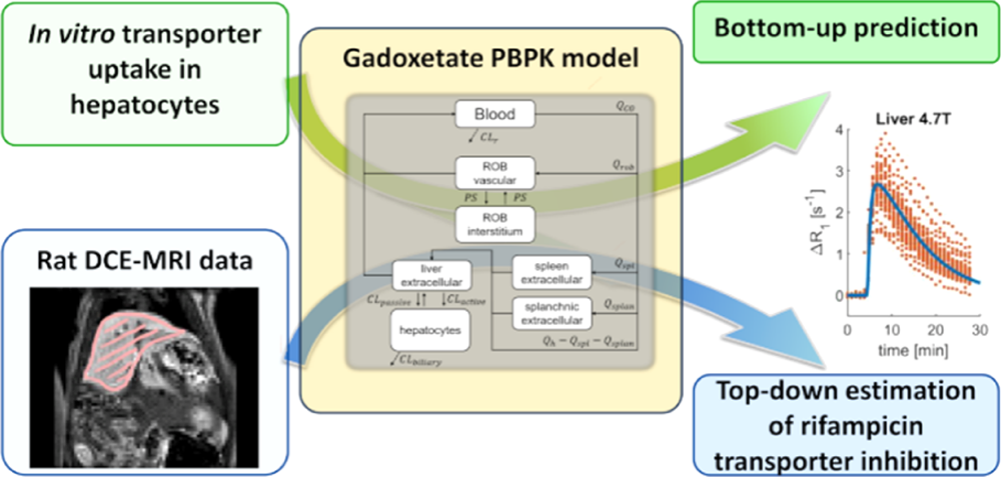

Physiologically based pharmacokinetic (PBPK) models are increasingly used in drug development to simulate changes in both systemic and tissue exposures that arise as a result of changes in enzyme and/or transporter activity. Verification of these model-based simulations of tissue exposure is challenging in the case of transporter-mediated drug–drug interactions (tDDI), in particular as these may lead to differential effects on substrate exposure in plasma and tissues/organs of interest. Gadoxetate, a promising magnetic resonance imaging (MRI) contrast agent, is a substrate of organic-anion-transporting polypeptide 1B1 (OATP1B1) and multidrug resistance-associated protein 2 (MRP2). In this study, we developed a gadoxetate PBPK model and explored the use of liver-imaging data to achieve and refine in vitro–in vivo extrapolation (IVIVE) of gadoxetate hepatic transporter kinetic data. In addition, PBPK modeling was used to investigate gadoxetate hepatic tDDI with rifampicin i.v. 10 mg/kg. In vivo dynamic contrast-enhanced (DCE) MRI data of gadoxetate in rat blood, spleen, and liver were used in this analysis. Gadoxetate in vitro uptake kinetic data were generated in plated rat hepatocytes. Mean (%CV) in vitro hepatocyte uptake unbound Michaelis–Menten constant (*K*_m,u_) of gadoxetate was 106 μM (17%) (*n* = 4 rats), and active saturable uptake accounted for 94% of total uptake into hepatocytes. PBPK–IVIVE of these data (bottom-up approach) captured reasonably systemic exposure, but underestimated the in vivo gadoxetate DCE–MRI profiles and elimination from the liver. Therefore, in vivo rat DCE–MRI liver data were subsequently used to refine gadoxetate transporter kinetic parameters in the PBPK model (top-down approach). Active uptake into the hepatocytes refined by the liver-imaging data was one order of magnitude higher than the one predicted by the IVIVE approach. Finally, the PBPK model was fitted to the gadoxetate DCE–MRI data (blood, spleen, and liver) obtained with and without coadministered rifampicin. Rifampicin was estimated to inhibit active uptake transport of gadoxetate into the liver by 96%. The current analysis highlighted the importance of gadoxetate liver data for PBPK model refinement, which was not feasible when using the blood data alone, as is common in PBPK modeling applications. The results of our study demonstrate the utility of organ-imaging data in evaluating and refining PBPK transporter IVIVE to support the subsequent model use for quantitative evaluation of hepatic tDDI.

## Introduction

The physiologically based pharmacokinetic (PBPK) modeling approach provides an effective mechanistic framework for quantitative translation of pharmacokinetic (PK) data. One of the highest impact areas of PBPK modeling is the prediction of drug–drug interactions (DDI). When performed using appropriately validated and refined models, PBPK modeling can support drug labeling and facilitate precision dosing in the absence of suitable clinical data.^1−3^ Regulatory impact of PBPK models so far is the highest for drugs that are either metabolized by, or are inhibitors of, hepatic and/or intestinal cytochrome P450 enzymes. Confidence is lower for PBPK models that involve drugs that are substrates or inhibitors of transporter proteins, such as hepatic organic anion transporter polypeptides (OATP).^4−7^ These trends are in part due to the additional complexity and uncertainty in the quantitative in vitro–in vivo extrapolation (IVIVE) of transporter kinetic data used to obtain drug-specific parameters of PBPK models.^1,4,8^ Moreover, for these drugs, the lack of in vivo tissue exposure data to support PBPK model development and verification of tissue simulations represents a key limitation.^4^

Direct measurement of in vivo drug concentration–time data in specific tissues of interest is practically and ethically challenging.^4^ However, an understanding of these local concentrations (total and unbound) can aid the delineation of sources of variability in drug response, for which measurements of drug concentrations in plasma may be insufficient.^4,6,9^ For drugs predominantly eliminated by liver, perturbations of efflux transporters relevant for their biliary excretion may lead to clinically relevant changes in liver exposure, which may not be reflected in the systemic concentrations (depending on rate-limiting processes).^4,10,11^ In this context, PBPK model-based predictions of local drug concentrations represent a useful surrogate, yet verifying key assumptions of model structure and parameter values (e.g*.,* efflux clearances) solely from plasma clinical data is challenging.

Application of imaging techniques such as positron emission tomography (PET), single-photon emission computed tomography (SPECT), and dynamic contrast-enhanced magnetic resonance imaging (DCE–MRI) enables the derivation of local tissue concentrations of radiolabeled compounds or contrast agents in vivo. Such techniques have shown promising results in delineating the roles of uptake and efflux transporters based on measurement of concentrations over time in the liver.^12−18^ An advantage of DCE–MRI over SPECT and PET is that study subjects are not exposed to ionizing radiation. In addition, DCE–MRI contrast agents are commonly used in clinical practice, do not require specialized synthesis facilities, and are, therefore, more easily accessed than PET or SPECT tracers.^18,19^

Gadoxetate is a metabolically stable MRI contrast agent currently indicated for detection and characterization of lesions in patients with known or suspected focal liver disease.^20^ This contrast agent has been shown to be a substrate for human uptake transporters OATP1B1, OATP1B3, and sodium/taurocholate co-transporting polypeptide and an efflux transporter multidrug resistance-associated protein 2 (MRP2). In addition, gadoxetate has been reported to be a substrate for rat Oatp1a1^21−25^ and Mrp2 based on in vivo studies on Mrp2-deficient rats.^26^ Trends in the literature indicate increasing interest in using gadoxetate for evaluating the liver transporter inhibition noninvasively.^4,13,27−29^ Researchers have continued to advance multicompartmental modeling for deriving quantitative parameters reflecting the liver transporter activity, using gadoxetate administered either alone or in combination with perpetrators of transporters relevant for gadoxetate disposition.^13,27−29^ PBPK modeling has been previously applied to DCE–MRI data with other gadolinium-based contrast agents than gadoxetate.^30,31^ Recently, a minimal PBPK model of gadoxetate in humans was reported, where gadoxetate systemic exposure and urinary data were used for model development.^32^

With the aim of evaluating gadoxetate as a potential imaging biomarker for hepatic transporter DDIs, we developed a reduced gadoxetate PBPK model for characterizing the PK of this imaging agent in rat blood, spleen, and liver and its interaction with a potent OATP1B inhibitor, rifampicin. Gadoxetate in vitro uptake kinetics was characterized over a concentration range in plated rat hepatocytes, and these data were implemented in the reduced PBPK model with mechanistic description of hepatobiliary disposition of gadoxetate. Although systemic exposure was predicted well, initial IVIVE (bottom-up approach) significantly underestimated in vivo gadoxetate DCE–MRI liver elimination. Subsequently, liver-imaging data of gadoxetate administered alone (control phase) were used to refine PBPK transporters kinetic parameters in a top-down manner. Finally, simultaneous fitting of DCE–MRI data from both the control and the rifampicin phases was performed (as per ref (33).) to determine the effect of rifampicin on the systemic and intrahepatic concentrations of gadoxetate, and to test whether the inclusion of the inhibitory phase data in the parameters’ identification would impact the estimated values based on the control phase. To the best of our knowledge, this is the first study that uses liver DCE–MRI data to refine the transporter IVIVE within the PBPK framework.

## Experimental Section

### In Vitro Uptake in Plated Rat Hepatocytes

Male adult Sprague-Dawley rats (Charles River, Margate, Kent, UK) were housed in groups of two in individually ventilated cages with free access to food (Chow rat and mouse diet) and fresh drinking water. The designated rat housing facility maintained a controlled temperature (20 ± 3 °C), humidity (40–70%), and 12 h light/dark cycle conditions. All animal protocols were approved by the University of Manchester review committee and adhered to the UK Home Office Animals (Scientific Procedures) Act (1986). Rats (250–300 g) were sacrificed using CO_2_ overdose followed by cervical dislocation in the morning of the study day. The kinetics experiment, as described below, was performed using *n* = 4 animals. A minimum of *n* = 3 was required to explore interanimal variability in kinetic parameters, with surplus hepatocytes also used from an additional animal that was part of a separate project. Primary rat hepatocytes, prepared as described below, were used to evaluate test compound cytotoxicity/effect on hepatocyte attachment (*n* = 3), preliminary assays to determine uptake assay conditions (*n* = 4), and for assays that were failed, for example, poor cell viability (*n* = 6 animals).

Isolation of hepatocytes using ex vivo collagenase perfusion was performed, followed by cell count and viability assessment, as previously described.^34−36^ Cell preparations with viability <85% were not used for experiments. Hepatocytes were seeded at 240,000 cells per well in collagen I-coated 24-well plates, and incubated for at least 2 h at 37 °C and 95% air/5% CO_2_ to allow cell attachment to the plate.^36^

Uptake experiments were performed at 37 °C with duplicate incubations per condition, as previously described.^35^ Uptake of gadoxetate (Primovist injection solution, Bayer, Germany) was evaluated following incubation (0.5–150 min) at nominal media concentrations of 0.01–10 mM. Extended incubation timepoints (up to 150 min) were selected based on previous publications^35^ and existing in-house data to enable characterization of the steady-state intracellular concentration. Uptake of pitavastatin (Sequoia Research Products Ltd, Pangbourne, UK) (0.2 μM) in the absence and presence of a pan-inhibitor of uptake transporters, rifamycin SV (Sigma-Aldrich, Poole, UK) (100 μM), was also evaluated for 0.5–2 min as positive control for functional transporter activity. Following sample preparation including addition of internal standard (Table S1), gadoxetate and pitavastatin in cell samples, and gadoxetate in media samples, were quantified by liquid chromatography–tandem mass spectrometry (LC–MS/MS). LC–MS/MS quantification was performed using selective reaction monitoring (SRM) against calibration standards; only standards within 30% of nominal concentration were included. LC–MS/MS equipment and conditions are listed in Table S1. The protein content of plated rat hepatocytes was measured using the Pierce BCA Protein Assay Kit (Thermo Fisher Scientific, Paisley, UK).

### Data Analysis and Quantitative Translation

In vitro hepatocyte uptake (i.e.*,* the amount of gadoxetate in cell as quantified by LC–MS/MS) (pmol) at each time point was normalized for cellularity using the measured protein content and assuming that 10^6^ hepatocytes contain 1 mg protein.^34^ For gadoxetate, the data from each animal were used for simultaneous estimation of in vitro kinetic parameters using a mechanistic hepatocyte model reported previously.^35^ It should be noted that CL_passive,u_ implies nonsaturable clearance equal in both directions under experimental conditions (0.01–10 mM).
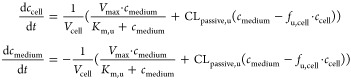
1where *K*_m,u_ represents the unbound Michaelis constant (μM); *V*_max_ the maximum transport rate (pmol/min/10^6^ cells); and *f*_u,cell_ the fraction unbound in cell and nonsaturable, bidirectional clearance (CL_passive,u_; μL/min/10^6^ cells). *c*_cell_ and *c*_medium_ represent concentrations in cell and medium compartments, respectively, and *V*_cell_ represents the cell volume.

Intracellular concentrations were calculated assuming a hepatocyte cell volume of 3.9 μL/10^6^ cells.^35^ Initial cell concentrations were estimated by back-extrapolation of the linear regression between time versus intracellular concentration for initial time-points (*t* ≤ 20 min) to *t* = 0 min. Measured media concentrations were similar to nominal concentrations, indicating negligible nonspecific binding, and therefore a nominal medium concentration was used as the initial condition for modeling. The model was implemented in MATLAB R2017a^37^ and the parameter estimation was performed using the *lsqnonlin* function.

Unbound intrinsic clearance (CL_active,u_; μL/min/10^6^ cells) of the saturable uptake for unbound concentrations ≪*K*_m,u_ was calculated using eq 2.
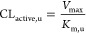
2

Unbound intrinsic uptake clearance (CL_int,u_; μL/min/10^6^ cells) of pitavastatin, in the absence and presence of rifamycin SV, was calculated from the uptake rate (*v*; pmol/min/10^6^ cells) and measured medium concentration (*c*_u_; μM). The uptake rate was obtained from the linear regression slope between the pitavastatin uptake amount (pmol) and time (min), normalized by the cell number.

IVIVE of gadoxetate CL_active,u_ and CL_passive,u_ was performed by scaling these parameters to in vivo values (CL_active,u,in vivo_ and CL_passive,u,in vivo_; mL/min/kg body weight) using a hepatocellularity of 120 × 10^6^ cells/g liver,^34^ and a liver weight (*w*_liver_) of 40 g/kg body weight,^38^ as in eq 3.
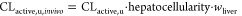
3

### DCE–MRI Dataset

DCE–MRI data generated using the 3D Fast Low Angle Shot RF-spoiled gradient echo sequence (FLASH) protocol in male Wistar-Han rats were used for assessing the PBPK IVIVE performances and for obtaining some of the PBPK parameters within the bottom-up and top-down approaches, respectively. The DCE–MRI data were acquired in a multicenter study at two magnetic field strengths, 4.7 and 7 T. Gadoxetate was administered at a dose of 25 μmol/kg, either alone or 1 h after IV administration of 10 mg/kg rifampicin. When gadoxetate was administered alone (control arm), 43 profiles of blood, spleen, and liver measured at a field strength of 4.7 T and 52 profiles of blood, spleen, and liver measured at 7 T were available. In the case of gadoxetate administered with rifampicin, 7 blood, spleen, and liver profiles at a field strength equal to 4.7 T and 6 at 7 T were available. All gadoxetate DCE–MRI data and study protocols are detailed in a companion paper (Hines et al. in submission).

The measured quantity in DCE–MRI is Δ*R*_1_ (s^–1^), the change of the water proton longitudinal relaxation rate, a magnetic property of the tissues, due to the presence of the contrast agent. In sufficiently homogeneous tissues, the tissue concentration of the contrast agent as a function of time, *c*(*t*), can be derived from Δ*R*_1_(*t*). The relationship between *c*(*t*) and Δ*R*_1_(*t*) depends on the physical interactions between the contrast agent molecules and the tissue.^39^ For a given tissue τ, the relation between Δ*R*_1,τ_(*t*) and *c*_τ_(*t*) is generally considered to be linear, as in eq 4.^39,40^
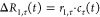
4

The proportionality constant *r*_1,τ_ (in L mmol^–1^ s^–1^) is the relaxivity of the contrast agent of the tissue τ.^39^ The *r*_1,τ_ values are typically difficult to measure in vivo, therefore, in this study, the ex vivo values in Table 1 were used, as per ref. 40. It has to be considered that *r*_1,τ_ and Δ*R*_1,τ_ change as a function of the magnetic field strength used by the magnetic resonance machine for image acquisition.^40^ Therefore, Δ*R*_1_ profiles acquired at different field strengths are not directly comparable.

**Table 1:** Ex Vivo Relaxivity (*r*_1_, [s^–1^/mM]) Values at Two Different Field Strengths^40^

tissues	4.7 T	7 T
blood^a^	6.4	6.2
hepatocytes	7.6	6

a Spleen *r*_1_ values were assumed to be equal to the blood.

### Gadoxetate PBPK Model

A reduced PBPK model was developed to describe the PK of gadoxetate in rats; a permeability-limited liver model was implemented capturing relevant processes, as done previously.^6,41^ The gadoxetate PBPK model is composed of seven compartments and its structure is shown in Figure 2. The compartments represent the blood, spleen, splanchnic organs, liver interstitial space, hepatocytes, and the rest of the body (ROB) vascular and extravascular spaces. The ROB compartment includes muscles, skin, bones, and fat among others. Details of model equations and physiological parameters values are reported in the Supporting Information, Sections 2, 3, and 5.

**Figure 1:**
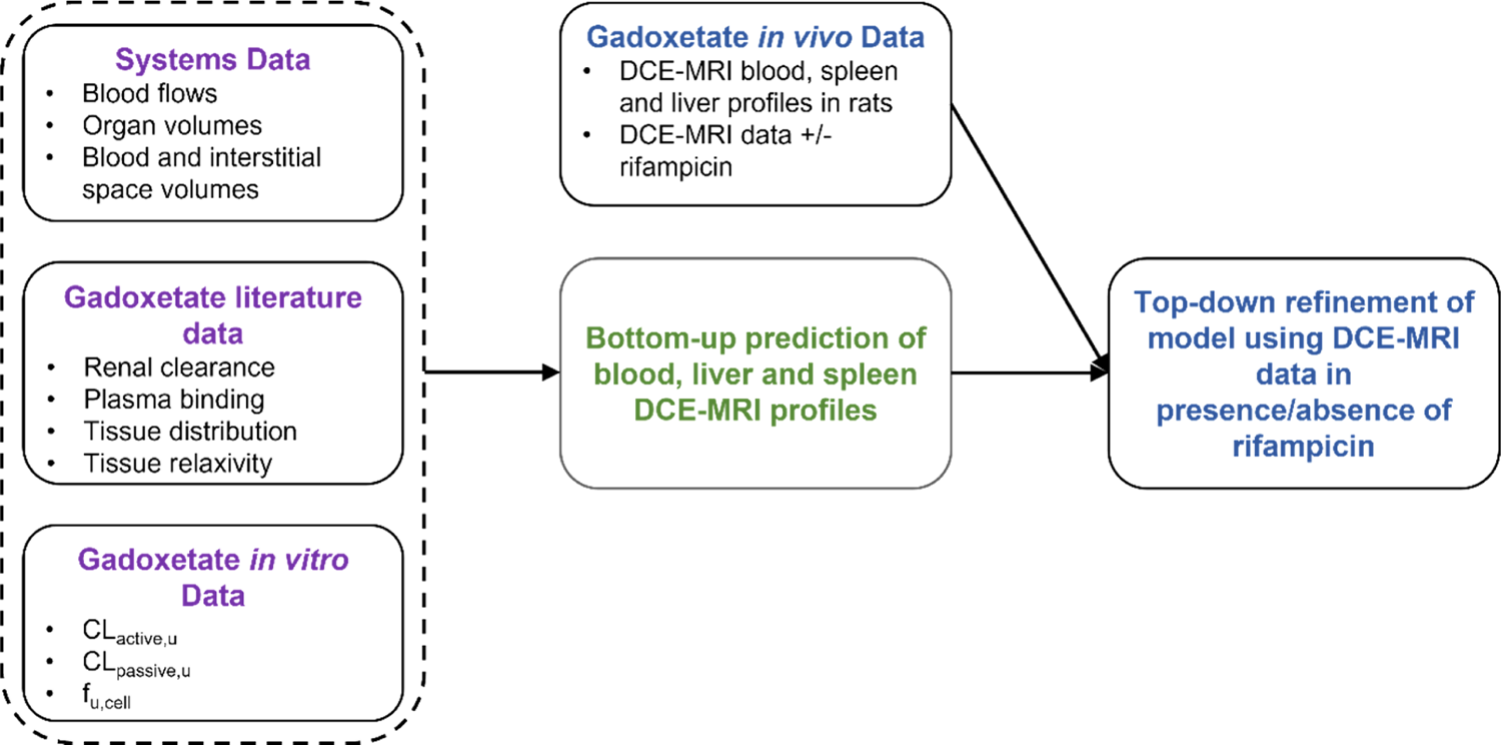
Development of PBPK model for gadoxetate in rats. Initially, gadoxetate blood, liver, and spleen DCE–MRI profiles were prospectively predicted using the literature and transporter kinetic in vitro data. Subsequently, the gadoxetate in vivo DCE–MRI data were used to refine the PBPK model and estimate transporter kinetic parameters both in the presence and the absence of rifampicin, a potent OATP1B inhibitor.

**Figure 2:**
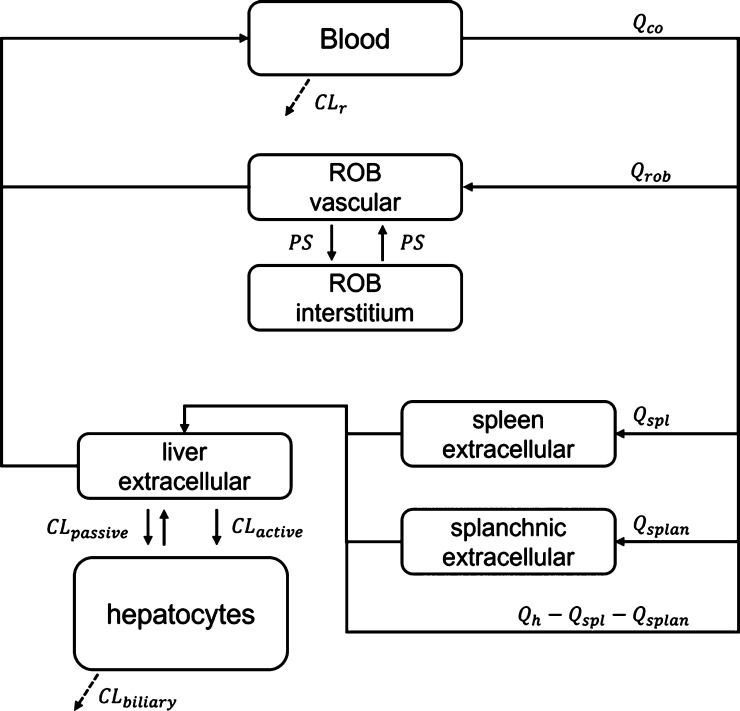
Structure of the reduced gadoxetate PBPK model. Continuous arrows represent the mass exchange within the system, while dashed arrows represent gadoxetate elimination. Subscripts *co*, *rob*, *spl*, *splan*, *h*, and *r* represent the cardiac output, ROB, spleen, splanchnic organs, hepatic, and renal, respectively. CL, Q, and PS represent the clearance processes, the blood flows, and the permeability surface product, respectively.

Standard kinetic models used for MRI contrast agents generally describe the organs by using three compartments: plasma, interstitial, and intracellular spaces.^12,42,43^ Gadoxetate distributes only in the extracellular space of all the organs; liver is the exception, where gadoxetate undergoes active uptake into the hepatocytes.^44^ Therefore, in the gadoxetate PBPK model, the volume of all the compartments, except the blood and the liver, corresponded to the organ extracellular space, that in turn was considered to be composed of the blood within the organ and the interstitial space. For highly vascularized and perfused organs with fenestrated capillaries (e.g., liver), the exchange between the plasma and the interstitial space is generally considered to be fast.^12,19^ Therefore, for liver, spleen, and splanchnic compartments, the extracellular volumes were considered to be the sum of the blood within the organ and the interstitial space volumes. However, this hypothesis does not hold true for all the organs.^12,43^ A permeability limitation between the vascular and interstitial space was, therefore, assumed for the ROB compartment, as shown in eq 5.
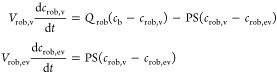
5*c*_b_, *c*_rob,v_ and *c*_rob,ev_ [μmol/L] are the gadoxetate concentration in blood and ROB vascular and extravascular compartments; *V*_rob,v_ and *V*_rob,ev_ [L] are the ROB vascular and extravascular compartment volumes, respectively; and PS [L/h] is the permeability surface product.

To describe the gadoxetate-active uptake into the hepatocytes, a permeability-limited liver model was used, as shown in eq 6.
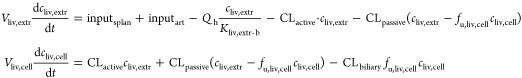
6input_splan_ [μmol/h] represent the venous input from the splanchnic organs, while input_art_ [μmol/h] is the input from the hepatic artery; *c*_liv,extr_ and *c*_liv,cell_ [μmol/L] are the drug concentrations in the extracellular liver (tissue blood plus interstitial space) and in the hepatocytes, respectively; *V*_liv,extr_ and *V*_liv,int_ [L] are the extracellular and hepatocytes liver volumes; *Q*_h_ [L/h] is the liver blood flow; *K*_liv,extr-b_ is the extracellular liver to blood partition coefficient; CL_active_ and CL_passive_ [L/h] are the active and passive clearances across the hepatocytes cell membrane; CL_biliary_ [L/h] is the clearance representing the excretion from the hepatocytes into the bile; and *f*_u,liv,cell_ is the gadoxetate fraction unbound in the hepatocytes (obtained from the in vitro generated data *f*_u,cell_). Recent PBPK studies of hepatic transporter substrates have used a 5-compartment liver model^45^ and the use of this particular liver model was also explored in the current PBPK modeling.

The DCE–MRI data used in this study were reported as Δ*R*_1_. Therefore, the relationship between the compartmental concentrations represented by the PBPK model state variables and the Δ*R*_1_ measurements for the blood, liver, and spleen needed to be described. The linear relationship in eq 4 is not valid for Δ*R*_1_ of the liver, Δ*R*_1,liv_, because intracellular and extracellular tissue compartments have different relaxivities. To derive Δ*R*_1,liv_, a volume fraction-weighted mean of the contributions of the gadoxetate concentration in all the compartments used to model the liver was performed.^40^ In the spleen, the entire distribution space was supposed to have the same relaxivity as blood and, therefore, eq 4 can be applied directly to derive spleen concentrations from Δ*R*_1,spl_. Concerning the blood, the blood value for *r*_1_ in Table 1 allows Δ*R*_1,b_ to be directly related to the blood concentration. The relations between Δ*R*_1,liv_, Δ*R*_1,spl_, and Δ*R*_1,b_ and the concentrations of the PBPK compartments are shown in eq 7.
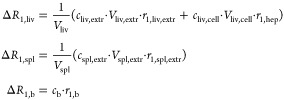
7*c*_spl,extr_ is the extracellular concentration in the spleen and *V*_spl,extr_ is the extracellular volume of the spleen and *V*_liv_ and *V*_spl_ are the whole volumes of liver and spleen. *r*_1,liv,extr_ and *r*_1,spl,extr_ were assumed to be equal to *r*_1,b_, as described in Hines et al.

Another difference of the DCE–MRI data relative to the drug concentration commonly used in PBPK modeling is that Δ*R*_1_ values do not correspond uniquely to a specific time point. In fact, each of the data are acquired during a time interval Δ*t*, which was 57 s in the current dataset. To account for this characteristic when performing the parameter estimation, the residuals were calculated as the difference of the observed Δ*R*_1_ corresponding to a given time interval minus the mean of the simulated PBPK Δ*R*_1_ within the same interval, as explained in the Supporting Information, Section 4.

### PBPK Analysis Overview: Bottom-Up, Top-Down, and Estimation of the Rifampicin Effect

The PBPK analysis was performed in three sequential steps: IVIVE of gadoxetate transporter kinetic data obtained in rat hepatocytes (bottom-up predictions), PBPK model refinement using DCE–MRI imaging data, and estimation of the transporter-mediated interaction with rifampicin (Figure 1).

All the parameters were initially obtained using literature values and in vitro experiments. Prospective transporter clearance IVIVE was performed with the aim of predicting the gadoxetate Δ*R*_1_ in blood, spleen, and liver after administration of gadoxetate alone (in the absence of an inhibitor). CL_active_, CL_passive_, and *f*_u,liv,cell_ were obtained from the in vitro experimental values obtained in this study, as detailed in the section “Data Analysis and Quantitative Translation”. CL_r_ was fixed to a literature value, as in Table 3. PS and CL_biliary_ could not be obtained from the in vitro experiments, and therefore the values for both parameters were assumed equal to CL_passive_. All the other parameters were obtained using literature values, as detailed in the Supporting Information, Sections 2 and 5.

An uncertainty analysis was performed to account for the in vitro data uncertainty within the bottom-up transporter clearance IVIVE.^46^ Briefly, in this analysis, all the uncertain or unknown parameters were considered as random variables with a given probability distribution function (pdf) and then a Monte Carlo simulation was performed. In the Monte Carlo simulations, the samples were extracted from the parameters’ joint pdf and, for each sample, the model was evaluated. The uncertain parameters considered in this analysis were: *V*_max_, *K*_m,u_, CL_passive_, *f*_u,liv,cell_, PS, and CL_biliary_. All these parameters were considered to be independent and uniformly distributed between the ranges reported in Table 2. A global sensitivity analysis (GSA) with the standardized regression coefficient (SRC) method^47,48^ was then performed considering the intracellular liver AUC calculated from 0 to 100 h after gadoxetate administration as the PK endpoint. The number of samples in both the uncertainty analysis and GSA was set to 10,000. The confidence intervals of the sensitivity indices were calculated by using 1,000 bootstrap samples.^49^

**Table 2:** Parameters Derived from the Mechanistic Modeling of Gadoxetate Kinetic In Vitro Data in Plated Rat Hepatocytes

	animal					
parameter	1	2	3	4	average	CV %
*V*_max_ [pmol/min/10^6^ cells]	350.4	370.9	221.7	368.8	327.95	22%
*K*_m,u_ [μM]	114.1	115.8	79.8	115.3	106.25	17%
CL_passive,u_ [μL/min/10^6^ cells]	0.091	0.202	0.274	0.203	0.193	39%
*f*_u,liv,cell_	0.759	0.709	0.418	0.704	0.648	24%
CL_active,u_^a^ [μL/min/10^6^ cells]	3.07	3.20	2.78	3.20	3.06	6%
maximum % active^b^	97	94	91	94	94	3
% passive^c^	3	6	9	6	6	41

a *V*_max_/*K*_m,u_.

b CL_active,u_/(CL_active,u_ + CL_passive,u_).

c CL_passive,u_/(CL_active,u_ + CL_passive,u_).

Concerning the top-down analysis, the Δ*R*_1_ blood, spleen, and liver profiles of the gadoxetate control group were used to refine the transporter IVIVE with the PBPK model. In this context, a naïve pooled approach was used for estimating CL_active_, CL_biliary_, and PS, while CL_passive_ and *f*_u,liv,cell_ were fixed to the in vitro values, and CL_r_ was fixed to the literature value, as reported in Table 3.

**Table 3:** Bottom-Up Scaled and Top-Down Estimated Parameters for Gadoxetate PBPK Model

parameter name	bottom-up scaled values	top-down estimates
CL_active_ [L/h]	0.23^a^	2.17 (11.5%)^b^
CL_biliary_ [L/h]	0.014^a^^,^^c^	0.07 (3.2%)^b^
PS [L/h]	0.014^a^^,^^c^	0.62 (6.3%)^b^
CL_passive_ [L/h]^d^	0.014	
*f*_u,liv,cell_^d^	0.648	
CL_r_ [L/h]^e^	0.17	

a Mean values calculated from the Monte Carlo analysis.

b Mean (CV %) of 1000 bootstrap samples.

c Value assumed equal to CL_passive_.

d Refers to the mean in vitro value in Table 2.

e Calculated as CL_total_ × *f*_e_, where CL_total_ is the total blood clearance, equal to 36.7 mL/min/kg and *f*_e_ is the fraction excreted in the urine, equal to 0.305.^44^ The rat weight was considered 0.25 kg.

The extent of interaction with rifampicin was estimated by performing a simultaneous fitting of gadoxetate Δ*R*_1_ in the control and inhibitory phase. In this analysis, PS was considered the same for both phases, whereas the other parameters were separately estimated in the absence (CL_active_ and CL_biliary_) and in the presence of rifampicin (CL_active,inh_ and CL_biliary,inh_). CL_biliary,inh_ was considered to account for possible inhibition of Mrp2 in rat by rifampicin, as previously reported.^45^ To understand the impact of the inclusion of the liver-imaging data on the parameter optimization, the simultaneous estimation was also performed considering only the blood data.

All the PBPK analyses were performed in MATLAB R2020a,^50^ the ordinary differential equations were solved with the function “*ode15s*”, while the parameter optimization was performed with the function “*lsqnonlin*”. The uncertainty of the parameters estimate was evaluated with the case-bootstrap,^51^ using 1000 samples.

## Results

### In Vitro Uptake of Gadoxetate in Plated Rat Hepatocytes

**Figure 3:**
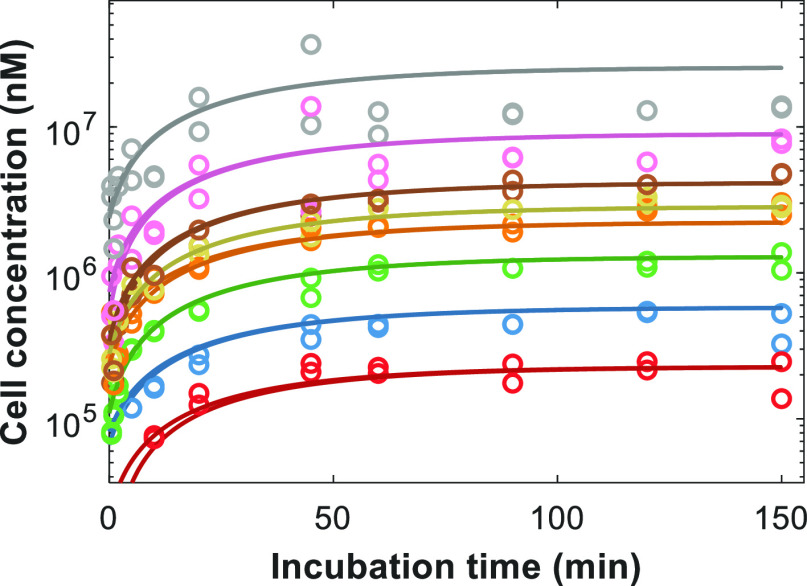
Representative example of fitting of mechanistic hepatocyte model to in vitro gadoxetate uptake data in plated rat hepatocytes from a single animal, with each nominal concentration run in duplicate. Colored lines and symbols represent simulated and observed data for experiments performed with nominal initial media concentrations of 10 μM (red), 30 μM (blue), 100 μM (green), 300 μM (orange), 500 μM (gold), 1 mM (brown), 3 mM (pink), and 10 mM (gray), respectively.

Gadoxetate exhibited concentration-dependent uptake into plated primary rat hepatocytes. In this analysis, concentrations in hepatocyte lysate that were below the lower limit of quantification (0.2 μM) were excluded; these excluded data typically represented the lower concentrations evaluated (e.g., 0.01–0.1 mM) and the earliest time points. Following calculation of the intracellular concentrations from lysate concentrations, the mechanistic hepatocyte model was used to estimate the in vitro hepatocyte uptake kinetic parameters (Figure 3). Gadoxetate *K*_m,u_ mean (% coefficient of variation, CV) was 106 μM (17%) for *n* = 4 animals, while *f*_u,cell_ was 0.65 (24%) (Table 2). Saturable active uptake was estimated to be the predominant process, with 94% contribution to total uptake. Unbound intrinsic uptake clearance of pitavastatin by plated rat hepatocyte in the absence of rifamycin SV, using same animals as gadoxetate experiments, was 95.7 μL/min/10^6^ cells (24%) and addition of 100 μM rifamycin SV reduced the uptake of pitavastatin by 54% (23%). The rank order of unbound active uptake clearance in the absence of an inhibitor for gadoxetate and pitavastatin was consistent, although there was lower inter animal variability in gadoxetate estimates (Figure S1). Translation of the in vitro uptake clearances of gadoxetate gave a predicted in vivo active uptake and passive clearances of 14.7 and 0.93 mL/min/kg body weight, respectively.

### Bottom-up PBPK Predictions of DCE–MRI Data in Rats

**Figure 4:**
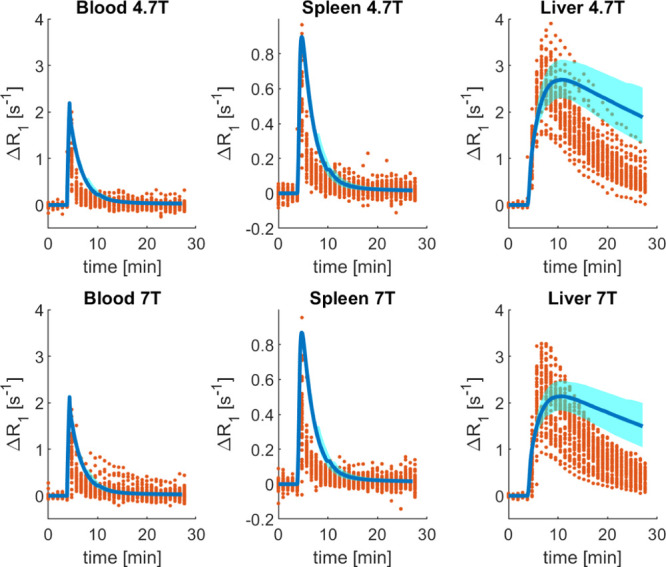
Comparison of the observed gadoxetate Δ*R*_1_ and prediction based on transporter IVIVE in the gadoxetate PBPK model. The red circles represent the individual data points of each rat [4.7 T: *n* = 33 animals from two sites; 7 T: *n* = 43 animals from two sites; and some animals were scanned twice (Hines et al. submitted)], the thick blue lines are the median of the PBPK predictions, and the cyan-shaded areas are the 95% confidence intervals. PBPK predictions and observed data vs time are reported for the rat blood, spleen, and liver Δ*R*_1_ at two field strengths, 4.7 T (top row) and 7 T (bottom row).

The results of the prospective bottom-up IVIVE of transporter kinetic data are shown in Figure 4, where the PBPK model predictions were compared to the observed gadoxetate Δ*R*_1_ profiles in blood, spleen, and liver at two field strengths, 4.7 and 7 T. The GSA results are shown in Figure S2.

The fast disappearance of gadoxetate from both the blood and the spleen in rat was reasonably predicted by the PBPK model, despite relatively minor overprediction of Δ*R*_1_ (Figure 4): mean predicted blood and spleen AUC values were up to 2.7-fold higher depending on the field strength used. In contrast, predicted liver concentration–time profiles by the gadoxetate PBPK model were not in agreement with the observed data and the overall dynamics was not captured well. In Figure 4, the cyan-shaded area represents the 95% confidence interval of the predictions, considering the uncertainty in selected parameters (*V*_max_, *K*_m,u_, CL_passive_, *f*_u,liv,cell_, PS, and CL_biliary_). For both blood and spleen, the parameter uncertainty had a minimal impact on the respective predicted Δ*R*_1_ profiles, as CV values for all the blood and spleen AUC at both the field strengths were lower than 11%. However, this was not the case for the liver AUC, where the CV was ∼32%.

For the GSA, the SRC method was used and the liver AUC was considered as the model output. The SRC method is suitable when the input–output (i.e.*,* uncertain model parameters–hepatocyte AUC) relationship is linear. In our case, the *R*^2^ of the linear regression is 0.93, therefore, the linearity condition was considered to be satisfied. In Figure S2, the squared standardized regression coefficients (SRC^2^) are reported. When the model is linear, the SRC^2^ correspond to the portion of output variance explained by the parameters, and thus they correspond to the first order effect of the variance-based GSA.^52^ The GSA showed that the most important parameters for explaining the liver AUC were CL_biliary_ and *f*_u,liv,cell_, whereas the uncertainties of CL_passive_, *V*_max_, and *K*_m,u_ (and consequently of CL_active_) had a minimal impact on the gadoxetate liver AUC variation.

### Refinement of Gadoxetate Transporter Kinetics in the PBPK Model Using Gadoxetate Liver-Imaging Data

**Figure 5:**
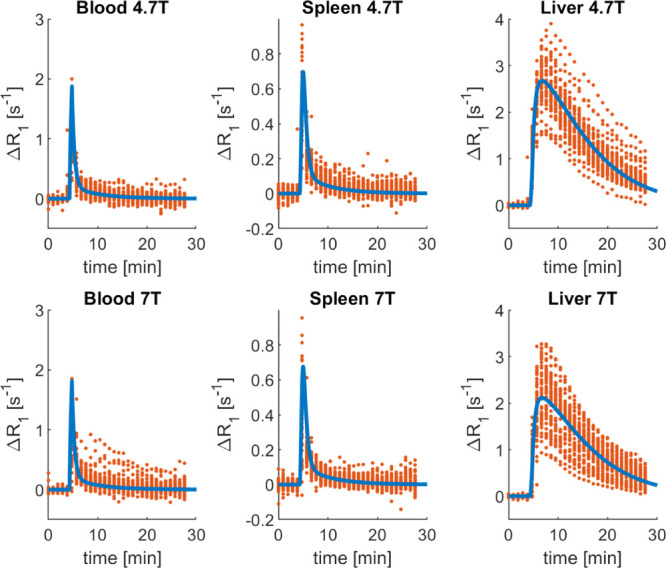
Results of the gadoxetate PBPK model after parameter estimation of selected parameters (CL_active_, CL_biliary_, and PS) to DCE–MRI data following administration of gadoxetate alone. The red dots represent the individual data points of each rat [4.7 T: *n* = 33 animals from 2 sites; 7 T: *n* = 43 animals from 2 sites; and some animals were scanned twice (Hines *et al.,* submitted)], the thick blue lines are the PBPK simulations following model fitting. PBPK simulation and observed data vs time are reported for the blood, spleen, and liver Δ*R*_1_ at two field strengths, 4.7 T (first row) and 7 T (second row).

In this analysis, CL_active_, CL_biliary_, and PS were estimated from fitting the PBPK model to the blood, spleen, and liver Δ*R*_1_ profiles (Table 3). The bootstrap results are shown in the Supporting Information, Figure S3. The model accurately fitted the data for all the organs at both field strengths, as illustrated in Figure 5. In addition, the simulated percentage of dose excreted in urine and bile was 17 and 83%, respectively. The gadoxetate concentrations in all the PBPK compartments are shown in the Supporting Information, Figure S4. The drug concentration in the interstitial ROB was lower with respect to those in all the other compartments, with the estimated value of PS being one order of magnitude lower than CL_active_. The CV of CL_active_ was below 12%, while CL_biliary_ and PS were estimated with a higher precision.

To explore whether the blood data alone were sufficiently informative to obtain the transporter kinetic parameters of the model (as generally available for standard PBPK analyses^4,53,54^), parameter estimation was also performed using only the blood data. In this context, CL_biliary_ was practically unidentifiable (CV > 1000%). Moreover, the estimates of CL_active_ and PS were 1.88 and 1.92 L/h, differed from those reported in Table 3. Although this analysis resulted in a good fit of blood and spleen profiles, the description of the liver Δ*R*_1_ was poor (Supporting Information, Section 6.3). In our analysis, gadoxetate CL_r_ was fixed to a literature reported value.^44^ As a further exercise, we attempted to simultaneously estimate this parameter, in addition to CL_active_, CL_passive_, CL_biliary_, and PS. In this context, CL_active_ was practically unidentifiable, with a CV higher than 1000% (Supporting Information, Section 6.4). Considering combined hepatic and renal elimination of gadoxetate, information on the urinary or biliary amounts excreted would be beneficial for a precise parameter’s identification and optimization of both CL_r_ and CL_active_.

### Evaluation of Gadoxetate Transporter-Mediated Interaction with Rifampicin

To estimate the effect of rifampicin on gadoxetate DCE–MRI profiles in blood and liver, a simultaneous estimation was performed using both the control and inhibitory phases and PS, CL_active_, CL_biliary_, and CL_active,inh_, CL_biliary,inh_ were estimated. The results of the parameter identification are reported in Table 4, the fitting results for the control and rifampicin treated group in Figures S9 and 6, and the bootstrap results in Figure S11. In this exercise, rifampicin inhibition of CL_active_ was estimated to be 96%. Due to the uncertainty in the CL_biliary_ estimates, it was difficult to conclude whether differences in the CL_biliary_ between control and rifampicin phase are significant under current experimental conditions. The CL_active_ and PS values obtained from the simultaneous estimation were slightly different from the estimates from top-down approach (Table 3), but there were no appreciable differences in the description of the data (Figure S9).

**Figure 6:**
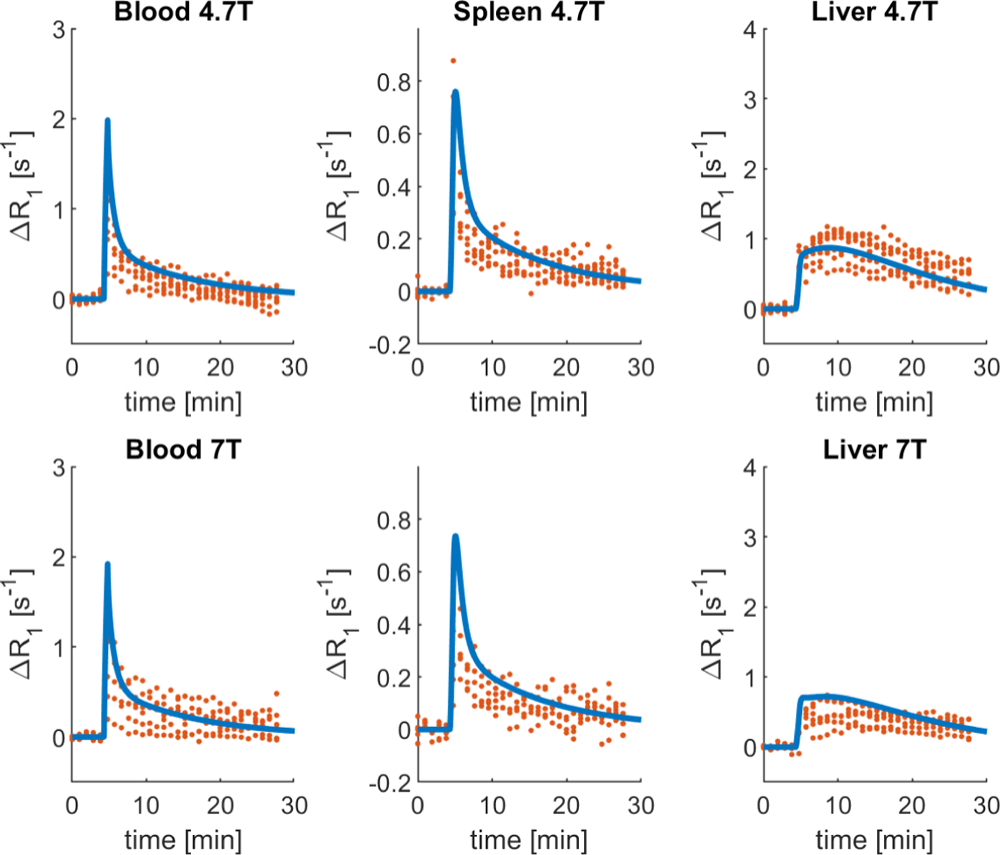
PBPK results of gadoxetate–rifampicin interaction following simultaneous estimation of gadoxetate Δ*R*_1_ profiles plus and minus rifampicin. The red dots refer to the individual data points of gadoxetate administered with rifampicin [4.7 T: *n* = 7 animals from 2 sites; 7 T: *n* = 6 animals from 2 sites (Hines et al.)] and the thick blue lines are the PBPK simulations following model fitting. PBPK simulations and observed data vs time are reported for the blood, spleen, and liver Δ*R*_1_ at two field strengths, 4.7 T (first row) and 7 T (second row).

**Table 4:** Gadoxetate Parameter Estimates Obtained by Simultaneous Fitting of the Data in the Control and Inhibitory (with Rifampicin) Phases

parameter name [L/h]	estimated value^a^ (control)	estimated value^a^ (with rifampicin)
CL_active_	2.38 (13.6%)	0.095 (16.1%)
CL_biliary_	0.07 (3.1%)	0.08 (16.7%)
PS	0.71 (5.7%)	

a Estimated value (CV), where CV (%) is the coefficient of variation of the estimates, calculated with 1000 bootstrap samples.

As a further analysis, the simultaneous estimation was performed considering only the blood Δ*R*_1_ profiles (Supporting Information, Section 6.6). In this analysis, both CL_biliary_ and CL_biliary,inh_ are practically unidentifiable (Table S5) and the liver profiles are not well predicted in both the control and inhibitory phases (Figures S12 and S13). In this analysis, CL_active_ and CL_active,inh_ were equal to 1.82 and 0.29 L/h. In particular, CL_active,inh_ resulted to be ∼3-fold higher than the one obtained when the liver profile was included in parameters identification (in Table 4). When considering only the blood Δ*R*_1_ profiles, an inhibition by rifampicin of 84% was estimated. However, considering the poor prediction of the liver data, in this case the extent of inhibition was likely to be underestimated.

It has been reported that PBPK liver models for certain OATP1B1 substrates (e.g*.,* pravastatin) best describe the PK data when the hepatocellular space is divided into five subcompartments.^55^ Therefore, the impact of different structures of liver model on gadoxetate parameter estimates was evaluated here by performing the simultaneous estimation with the 5-compartment liver model (Supporting Information, Section 7). The values of gadoxetate CL_active_ and CL_active,inh_ obtained using this approach were of the same order of magnitude with respect to the ones of the standard permeability-limited liver model, with no appreciable differences in the in vivo data fitting and in the extent of CL_active_ inhibition by rifampicin (Table S6, Figures S15 and S16).

## Discussion

Conventional PK DDI studies on drug development evaluate changes in drug exposure based on central plasma or blood concentrations, and may, therefore, be limited when pharmacological effects are driven by drug exposure in specific tissue or cells, as seen in the example of metformin DDIs.^56^ This issue is particularly evident when modulation of transporters (e.g*.,* in case of DDI) may cause different effects on drug exposure in the plasma and tissues of interest.^4^ PBPK modeling provides a mechanistic insight into the interplay of multiple processes at the tissue level and allows prospective prediction of transporter-mediated changes in tissue exposure. However, verification of such model-based simulations is challenged by the lack of appropriate tissue data in human or reliance on plasma clinical data that may not always be informative for PBPK model development and qualification.^4,41,53,54^ As such, imaging biomarkers for in vivo hepatobiliary transporter DDI are needed, enabling more ethical and efficient sampling of tissue concentrations of the transporter substrate than more invasive approaches (e.g., biopsy or sacrificial sampling done in preclinical species). Gadoxetate has been proposed as a potential imaging biomarker for evaluation of DDI mediated by OATP1B1 and MRP2.^12,13,57^ In this work, a PBPK model for the MRI contrast agent gadoxetate was developed to enable characterization of liver transporter DDI and to explore the use of liver-imaging data to achieve and refine hepatic transporter IVIVE.

### Advantages of Liver-Imaging Data for the Evaluation of PBPK IVIVE

Gadoxetate active and passive uptakes were characterized in vitro in plated rat hepatocytes and these data were used for prospective IVIVE of its transporter-mediated hepatic disposition. PBPK model predictions have captured reasonably well the observed Δ*R*_1_ blood and spleen profiles (Figure 4), but the liver data were poorly predicted. This unsatisfactory prediction of the in vivo profiles is most likely due to underprediction of CL_active_ (see Table 3) and lack of appropriate CL_biliary_ in vitro data. IVIVE of transporter kinetics has been reported to result in underprediction of in vivo hepatic clearance and plasma PK,^4,58^ but also of rosuvastatin active uptake clearance measured by PET imaging of the liver.^59^ Differences in transporter protein abundance between the cultured cells in vitro and liver tissue have been identified as one of the contributing factors to such underpredictions, highlighting the challenges remaining for transporter IVIVE.^4,58^ The current study highlighted the benefit of DCE–MRI liver data for the assessment of transporter-IVIVE performance. In classical IVIVE settings, model predictions are usually compared with plasma concentrations and the PK of the drugs investigated in the liver and other organs typically remains unknown.

The GSA performed here highlighted that the most important parameters in driving the gadoxetate liver AUC were CL_biliary_ and *f*_u,liv,cell_ (Figure S2). The importance of CL_biliary_ and *f*_u,liv,cell_ on liver AUC is completely expected from a PK point of view, as both parameters drive the removal of gadoxetate from the hepatocytes (eq 6). In contrast, variation in gadoxetate CL_active_ did not cause an appreciable effect on the liver AUC, in line with the understanding of the rate-limiting processes affecting its liver disposition. These results are in accordance with other examples of OATP1B substrates (e.g*.,* simvastatin),^6^ which are predominantly eliminated by the liver and where metabolic clearance/biliary excretion drives liver AUC rather than active uptake clearance.^4^

### Advantages of Using Liver-Imaging Data for Top-Down Refinements of PBPK IVIVE

In the top-down approach, observed blood, spleen, and liver Δ*R*_1_ profiles were used to refine the PBPK transporter parameters and to quantify the magnitude of gadoxetate DDI with rifampicin. Rifampicin single dose is clinically used as an OATP1B inhibitor^33^ for evaluation of DDI via this transporter.^60^ The current study aimed to develop and evaluate the PBPK model for gadoxetate; prospective prediction of the gadoxetate–rifampicin interaction was not performed due to uncertainties associated with IVIVE of in vitro inhibition data and complexities of substrate-dependent inhibition associated with OATP1B1.^58^ Application of the PBPK modeling for quantitative and translational prediction of gadoxetate–drug interactions will be explored in future work with an extended dataset of transporter inhibitors.

The optimized PBPK model accurately described the PK of gadoxetate in all the observed organs both in the control and in the inhibitory phase (Figures 5 and 6). Liver and blood DCE–MRI data when gadoxetate was administered alone were sufficient to obtain and refine gadoxetate transporter kinetic parameters in the PBPK model, resulting in CL_active_ that was one order of magnitude higher than the one predicted in the bottom-up manner from the in vitro data. Moreover, in the control phase, the simulated maximum gadoxetate concentration in the interstitial ROB was one order of magnitude lower than the predicted value for the hepatocytes (Figure S4). This suggests that, according to the PBPK model, in rats, gadoxetate distributes mainly into the hepatocytes and to a lower extent in the interstitial space of the other organs, in agreement with recent analysis of gadoxetate PK in humans.^32^

Use of both control and data obtained in the presence of rifampicin for simultaneous estimation resulted in comparable gadoxetate parameter estimates for the control phase (Table 4) to the ones estimated by using the DCE–MRI data of gadoxetate administered alone (Table 3). These results suggest that availability of liver-imaging data (in addition to blood) in the control phase alone was sufficient to appropriately characterize the activity of hepatic transporters involved in gadoxetate hepatic disposition. In cases when tissue data are not available, availability of clinical data reflecting perturbations of transporter mechanisms is crucial. For example, a recent population PK study of coproporphyrin (an endogenous biomarker for OATP1B-mediated DDI) highlighted that the availability of its clinical data in plasma and urine, both in the absence and presence of rifampicin, was crucial for the identifiability of its hepatic and renal elimination.^33^

Simultaneous fitting of the control and inhibition phase estimated that rifampicin causes an almost complete inhibition (96%) of the active uptake of gadoxetate into the hepatocytes. This result is in accordance with literature reports of interaction between rifampicin and OATP1B1 substrates.^61−64^ The PBPK model described very well the liver profiles of the 4.7 T group, but slightly overpredicted the 7 T group (Figure S10). The liver data of the inhibitory phase were quite noisy, sampling did not capture the full terminal phase of the liver profile (Figure 6), and the number of animals was lower with respect to the control group. All these factors may have contributed to higher uncertainty in CL_biliary_ identification, which was evident for the rifampicin phase (CV 16.7% relative to control phase CV 3.1%, Table 4). Studies on mrp2-deficient animals provide evidence of involvement of this transporter in biliary elimination of gadoxetate,^21,65^ supported also by some DCE–MRI studies, where a modest change in estimated efflux rates of gadoxetate from the liver was noted in the presence of rifampicin.^13^ The analysis indicated that a longer time scan in the liver would probably be beneficial for improved characterization of CL_biliary_ and its variability, especially in the rifampicin-treated group.

In the top-down analysis, the liver Δ*R*_1_ profiles played an essential role in the parameter identifiability. As expected, it was not possible to estimate the biliary clearance without including the information of gadoxetate PK in the liver^4^ (Figures S5, S12 and S13). Moreover, when fitting the model to only blood data, the extent of the active uptake inhibition was underestimated (96% inhibition estimated with liver data vs 84% without liver data) and the estimates of the active clearance and permeability surface product were different than when considering the liver profiles as well. Imaging methods can be a solution to characterize noninvasively organ concentrations, and thus can be particularly informative in the evaluation of DDIs via modulation of multiple transporters and/or refinement of PBPK modeling of tissue exposure. It is important to consider that gadoxetate has a substantial contribution of renal excretion to the overall elimination from the blood. Therefore, care should be taken in extending the results to drugs whose systemic exposure is mainly sensitive to modulation of liver active uptake.

### Technical Considerations of the Gadoxetate PBPK Model

Previous PBPK studies of hepatic transporter substrates have used a 5-compartment liver model, based on the empirical observation that this approach mimics the dispersion model.^45,55^ In the current analysis, we explored the use of a 5-compartment liver model for gadoxetate PBPK analysis and found a minimal impact on the description of the data and the estimation of DDI (see Supporting Information, Section 7).

The estimation of the PBPK model parameters in the current study used a naïve pooled data analysis approach. Such an approach lacks insights into interanimal and intersite variability, which would be required to give context to the estimated rifampicin treatment effects on transporter activities. As such, application of the PBPK model within a nonlinear mixed effect statistical framework should be considered for future research.

### Challenges in Using DCE–MRI Data within PBPK Modeling and the Simulation Framework

The use of the DCE–MRI data within PBPK modeling and simulations is not trivial. Perhaps, the most relevant issue that we have faced in using the DCE–MRI data within PBPK modeling was the uncertainty in Δ*R*_1_ profiles. As briefly outlined in the section “DCE–MRI dataset”, Δ*R*_1_ is derived from measured MRI signals using signal models that represent approximations of reality and depend on technical parameters that may not be known accurately—such as the flip angle of the MRI pulse sequence. These effects are known to cause some bias in the generated Δ*R*_1_ profiles,^66^ though this is continually being improved by better controlled acquisitions and refined signal models. The reproducibility of the DCE–MRI data used in this study was assessed across different sites and the technical parameters were chosen carefully after extensive optimization (Hines et al.). In our preliminary analyses, the suboptimal choice of some of these parameters resulted in discrepancies between the Δ*R*_1_ values of the two field strengths, and subsequent inability of the PBPK model to appropriately describe all the Δ*R*_1_ profiles. Similar to DCE–MRI, quantitative PBPK analysis of other imaging-derived PK data requires a signal conversion, whose accuracy in deriving the true concentration of an underlying tracer or contrast agent may differ depending on the maturity of the field and characteristics of the imaging technique (e.g*.,* a photon attenuation correction factor was required for quantitative PBPK analysis of ^99m^Tc-mebrofenin, an OATP1B/MRP2 substrate and a scintigraphic imaging agent^67^). In conclusion, we recommend the PBPK analysts dealing with the DCE–MRI data not to ignore the process of data generation and involve imaging experts in the modeling team.

## Conclusions

The current work illustrates the essential role of liver-imaging data/PK in the evaluation of predictive performance of prospective transporter IVIVE of gadoxetate within the PBPK framework. Moreover, the liver data were essential in refining the gadoxetate transporter IVIVE to appropriately describe organ concentrations and to adequately characterize the magnitude of hepatic transporter DDI with rifampicin. The use of the tissue exposure data for such purposes is still very limited. The analysis performed here provides novel insights that would be of particular importance for drugs with combined elimination (hepatic and renal, as in the case of gadoxetate), where the effects on the OATP1B1 uptake transporter may not be solely/easily deduced from the changes in the systemic exposure data. The results of this work highlight that gadoxetate is a promising probe to quantify the effect of perpetrator drugs on hepatic transporter (OATP1B and, potentially, MRP2) function in vivo. Work is ongoing to evaluate the performance of this imaging biomarker against OATP1B/MRP2 inhibitors with different degrees of potency and to extend the work into human.

## Supporting Information Available

In vitro uptake of gadoxetate in plated rat hepatocytes; PBPK model equations and residual calculations; derivation of the partition coefficients; calculation of the residuals; PBPK model parameters; PBPK results; impact of the number of liver compartments (PDF)

## Abbreviations

ΔR1: change in the relaxation rate
AUC: area under the concentration–time curve
CV: coefficient of variability
DCE: dynamic contrast enhanced
DDI: drug–drug interaction
GSA: global sensitivity analysis
LC–MS/MS: liquid chromatography–tandem mass spectrometry
MRI: magnetic resonance imaging
MRP: multidrug resistance-associated protein
OATP: organic-anion-transporting polypeptide
PBPK: physiologically based pharmacokinetic
pdf: probability density function
PK: pharmacokinetic
SRC: standardized regression coefficients
SRM: selective reaction monitoring
tDDI: transporter mediated drug–drug interaction

## References

[ref1] Grimstein M.; Yang Y.; Zhang X.; Grillo J.; Huang S.-M.; Zineh I.; Wang Y. Physiologically Based Pharmacokinetic Modeling in Regulatory Science: An Update From the U.S. Food and Drug Administration’s Office of Clinical Pharmacology. J. Pharm. Sci. 2019, 108, 21–25. 10.1016/j.xphs.2018.10.033. 30385284

[ref2] Darwich A. S.; Polasek T. M.; Aronson J. K.; Ogungbenro K.; Wright D. F. B.; Achour B.; Reny J.-L.; Daali Y.; Eiermann B.; Cook J.; Lesko L.; McLachlan A. J.; Rostami-Hodjegan A. Model-Informed Precision Dosing: Background, Requirements, Validation, Implementation, and Forward Trajectory of Individualizing Drug Therapy. Annu. Rev. Pharmacol. 2021, 61, 225 10.1146/annurev-pharmtox-033020-113257. 33035445

[ref3] Zhang X.; Yang Y.; Grimstein M.; Fan J.; Grillo J. A.; Huang S.-M.; Zhu H.; Wang Y. Application of PBPK Modeling and Simulation for Regulatory Decision Making and Its Impact on US Prescribing Information: An Update on the 2018-2019 Submissions to the US FDA’s Office of Clinical Pharmacology. J. Clin. Pharmacol. 2020, 60, S160–S178. 10.1002/jcph.1767. 33205429

[ref4] Guo Y.; Chu X.; Parrott N. J.; Brouwer K. L. R.; Hsu V.; Nagar S.; Matsson P.; Sharma P.; Snoeys J.; Sugiyama Y.; Tatosian D.; Unadkat J. D.; Huang S.-M.; Galetin A. Advancing Predictions of Tissue and Intracellular Drug Concentrations Using In Vitro, Imaging and Physiologically Based Pharmacokinetic Modeling Approaches. Clin. Pharmacol. Ther. 2018, 104, 865–889. 10.1002/cpt.1183. 30059145 PMC6197917

[ref5] Galetin A.; Zhao P.; Huang S.-M. Physiologically Based Pharmacokinetic Modeling of Drug Transporters to Facilitate Individualized Dose Prediction. J. Pharm. Sci. 2017, 106, 2204–2208. 10.1016/j.xphs.2017.03.036. 28390843

[ref6] Tsamandouras N.; Dickinson G.; Guo Y.; Hall S.; Rostami-Hodjegan A.; Galetin A.; Aarons L. Development and Application of a Mechanistic Pharmacokinetic Model for Simvastatin and Its Active Metabolite Simvastatin Acid Using an Integrated Population PBPK Approach. Pharm. Res. 2015, 32, 1864–1883. 10.1007/s11095-014-1581-2. 25446771

[ref7] Gertz M.; Cartwright C. M.; Hobbs M. J.; Kenworthy K. E.; Rowland M.; Houston J. B.; Galetin A. Cyclosporine Inhibition of Hepatic and Intestinal CYP3A4, Uptake and Efflux Transporters: Application of PBPK Modeling in the Assessment of Drug-Drug Interaction Potential. Pharm. Res. 2013, 30, 761–780. 10.1007/s11095-012-0918-y. 23179780

[ref8] Yoshida K.; Zhao P.; Zhang L.; Abernethy D. R.; Rekić D.; Reynolds K. S.; Galetin A.; Huang S.-M. In Vitro–In Vivo Extrapolation of Metabolism- and Transporter-Mediated Drug–Drug Interactions—Overview of Basic Prediction Methods. J. Pharm. Sci. 2017, 106, 2209–2213. 10.1016/j.xphs.2017.04.045. 28456729

[ref9] Rostami-Hodjegan A. Response to “The Link Between Pharmacodynamics and Physiologically Based Pharmacokinetic Models.”. Clin. Pharmacol. Ther. 2013, 93, 152 10.1038/clpt.2012.216. 23281422

[ref10] Köck K.; Brouwer K. L. R. A Perspective on Efflux Transport Proteins in the Liver. Clin. Pharmacol. Ther. 2012, 92, 599–612. 10.1038/clpt.2012.79. 22948894 PMC3725336

[ref11] Chu X.; Korzekwa K.; Elsby R.; Fenner K.; Galetin A.; Lai Y.; Matsson P.; Moss A.; Nagar S.; Rosania G. R.; Bai J. P. F.; Polli J. W.; Sugiyama Y.; Brouwer K. L. R. Intracellular Drug Concentrations and Transporters: Measurement, Modeling, and Implications for the Liver. Clin. Pharmacol. Ther. 2013, 94, 126–141. 10.1038/clpt.2013.78. 23588320 PMC3898878

[ref12] Ulloa J. L.; Stahl S.; Yates J.; Woodhouse N.; Kenna J. G.; Jones H. B.; Waterton J. C.; Hockings P. D. Assessment of Gadoxetate DCE-MRI as a Biomarker of Hepatobiliary Transporter Inhibition. NMR Biomed. 2013, 26, 1258–1270. 10.1002/nbm.2946. 23564602 PMC3817526

[ref13] Karageorgis A.; Lenhard S. C.; Yerby B.; Forsgren M. F.; Liachenko S.; Johansson E.; Pilling M. A.; Peterson R. A.; Yang X.; Williams D. P.; Ungersma S. E.; Morgan R. E.; Brouwer K. L. R.; Jucker B. M.; Hockings P. D. A Multi-Center Preclinical Study of Gadoxetate DCE-MRI in Rats as a Biomarker of Drug Induced Inhibition of Liver Transporter Function. PloS One 2018, 13, e0197213 10.1371/journal.pone.0197213. 29771932 PMC5957399

[ref14] Takashima T.; Kitamura S.; Wada Y.; Tanaka M.; Shigihara Y.; Ishii H.; Ijuin R.; Shiomi S.; Nakae T.; Watanabe Y.; Cui Y.; Doi H.; Suzuki M.; Maeda K.; Kusuhara H.; Sugiyama Y.; Watanabe Y. PET Imaging–Based Evaluation of Hepatobiliary Transport in Humans with (15R)-11C-TIC-Me. J. Nucl. Med. 2012, 53, 741–748. 10.2967/jnumed.111.098681. 22499612

[ref15] Takashima T.; Hashizume Y.; Katayama Y.; Murai M.; Wada Y.; Maeda K.; Sugiyama Y.; Watanabe Y. The Involvement of Organic Anion Transporting Polypeptide in the Hepatic Uptake of Telmisartan in Rats: PET Studies with [11C]Telmisartan. Mol. Pharm. 2011, 8, 1789–1798. 10.1021/mp200160t. 21812443

[ref16] Kaneko K.-i.; Tanaka M.; Ishii A.; Katayama Y.; Nakaoka T.; Irie S.; Kawahata H.; Yamanaga T.; Wada Y.; Miyake T.; Toshimoto K.; Maeda K.; Cui Y.; Enomoto M.; Kawamura E.; Kawada N.; Kawabe J.; Shiomi S.; Kusuhara H.; Sugiyama Y.; Watanabe Y. A Clinical Quantitative Evaluation of Hepatobiliary Transport of [11C]Dehydropravastatin in Humans Using Positron Emission Tomography. Drug Metab. Dispos. 2018, 46, 719–728. 10.1124/dmd.118.080408. 29555827

[ref17] He J.; Yu Y.; Prasad B.; Link J.; Miyaoka R. S.; Chen X.; Unadkat J. D. PET Imaging of Oatp-Mediated Hepatobiliary Transport of [11C] Rosuvastatin in the Rat. Mol. Pharm. 2014, 11, 2745–2754. 10.1021/mp500027c. 24957348

[ref18] Hernández Lozano I.; Bauer M.; Wulkersdorfer B.; Traxl A.; Philippe C.; Weber M.; Häusler S.; Stieger B.; Jäger W.; Mairinger S.; Wanek T.; Hacker M.; Zeitlinger M.; Langer O. Measurement of Hepatic ABCB1 and ABCG2 Transport Activity with [11C]Tariquidar and PET in Humans and Mice. Mol. Pharm. 2020, 17, 316–326. 10.1021/acs.molpharmaceut.9b01060. 31790256

[ref19] Sommer W. H.; Sourbron S.; Huppertz A.; Ingrisch M.; Reiser M. F.; Zech C. J. Contrast Agents as a Biological Marker in Magnetic Resonance Imaging of the Liver: Conventional and New Approaches. Abdom. Imag. 2012, 37, 164–179. 10.1007/s00261-011-9734-9. 21516381

[ref20] U.S. Food and Drug Administration. Drug Labeling-Package Insert: EOVIST (Gadoxetate Disodium) Injection [FDA Application No, (NDA) 022090] (accessed 20 Oct 2020).

[ref21] Jia J.; Puls D.; Oswald S.; Jedlitschky G.; Kühn J. P.; Weitschies W.; Hosten N.; Siegmund W.; Keiser M. Characterization of the Intestinal and Hepatic Uptake/Efflux Transport of the Magnetic Resonance Imaging Contrast Agent Gadolinium-Ethoxylbenzyl-Diethylenetriamine-Pentaacetic Acid. Invest. Radiol. 2014, 49, 78–86. 10.1097/rli.0b013e3182a70043. 24056116

[ref22] Jia J.; Keiser M.; Nassif A.; Siegmund W.; Oswald S. A LC–MS/MS Method to Evaluate the Hepatic Uptake of the Liver-Specific Magnetic Resonance Imaging Contrast Agent Gadoxetate (Gd-EOB-DTPA) in Vitro and in Humans. J. Chromatogr. B: Anal. Technol. Biomed. Life Sci. 2012, 891-892, 20–26. 10.1016/j.jchromb.2012.02.014. 22391331

[ref23] Nassif A.; Jia J.; Keiser M.; Oswald S.; Modess C.; Nagel S.; Weitschies W.; Hosten N.; Siegmund W.; Kühn J.-P. Visualization of Hepatic Uptake Transporter Function in Healthy Subjects by Using Gadoxetic Acid–Enhanced MR Imaging. Radiology 2012, 264, 741–750. 10.1148/radiol.12112061. 22771883

[ref24] van Montfoort J. E.; Stieger B.; Meijer D. K.; Weinmann H. J.; Meier P. J.; Fattinger K. E. Hepatic Uptake of the Magnetic Resonance Imaging Contrast Agent Gadoxetate by the Organic Anion Transporting Polypeptide Oatp1. J. Pharmacol. Exp. Ther. 1999, 290, 153–157. 10381771

[ref25] Leonhardt M.; Keiser M.; Oswald S.; Kühn J.; Jia J.; Grube M.; Kroemer H. K.; Siegmund W.; Weitschies W. Hepatic Uptake of the Magnetic Resonance Imaging Contrast Agent Gd-EOB-DTPA: Role of Human Organic Anion Transporters. Drug Metab. Dispos. 2010, 38, 1024–1028. 10.1124/dmd.110.032862. 20406852

[ref26] Muhler A.; Oude Elferink R. P. J.; Weinmann H.-J. Complete Elimination of the Hepatobiliary Mr Contrast Agent Gd-EOB-DTPA in Hepatic Dysfunction: An Experimental Study Using Transport-Deficient, Mutant Rats. Magma Magn. Reson. Mater. Phys. Biol. Med. 1993, 1, 134–139. 10.1007/bf01769415.

[ref27] Giraudeau C.; Leporq B.; Doblas S.; Lagadec M.; Pastor C. M.; Daire J.-L.; Van Beers B. E. Gadoxetate-Enhanced MR Imaging and Compartmental Modelling to Assess Hepatocyte Bidirectional Transport Function in Rats with Advanced Liver Fibrosis. Eur. Radiol. 2017, 27, 1804–1811. 10.1007/s00330-016-4536-7. 27553933

[ref28] Forsgren M. F.; Leinhard O. D.; Dahlström N.; Cedersund G.; Lundberg P. Physiologically Realistic and Validated Mathematical Liver Model Revels Hepatobiliary Transfer Rates for Gd-EOB-DTPA Using Human DCE-MRI Data. PloS One 2014, 9, e95700 10.1371/journal.pone.0095700. 24748411 PMC3991717

[ref29] Georgiou L.; Penny J.; Nicholls G.; Woodhouse N.; Blé F.-X.; Hubbard Cristinacce P. L.; Naish J. H. Quantitative Assessment of Liver Function Using Gadoxetate-Enhanced Magnetic Resonance Imaging: Monitoring Transporter-Mediated Processes in Healthy Volunteers. Invest. Radiol. 2017, 52, 111–119. 10.1097/rli.0000000000000316. 28002117 PMC5228626

[ref30] Spanakis M.; Marias K. In Silico Evaluation of Gadofosveset Pharmacokinetics in Different Population Groups Using the Simcyp Simulator Platform. In Silico Pharmacol. 2014, 2, 2 10.1186/s40203-014-0002-x. 27502621 PMC4644137

[ref31] Spanakis M.; Kontopodis E.; Van Cauter S.; Sakkalis V.; Marias K. Assessment of DCE–MRI Parameters for Brain Tumors through Implementation of Physiologically–Based Pharmacokinetic Model Approaches for Gd-DOTA. J. Pharmacokinet. Pharmacodyn. 2016, 43, 529–547. 10.1007/s10928-016-9493-x. 27647272

[ref32] Weiss M.; Siegmund W. Unusual Distribution Kinetics of Gadoxetate in Healthy Human Subjects Genotyped for OATP1B1: Application of Population Analysis and a Minimal Physiological-Based Pharmacokinetic Model. J. Clin. Pharmacol. 2021, 61, 506 10.1002/jcph.1762. 33084108

[ref33] Barnett S.; Ogungbenro K.; Ménochet K.; Shen H.; Lai Y.; Humphreys W. G.; Galetin A. Gaining Mechanistic Insight Into Coproporphyrin I as Endogenous Biomarker for OATP1B-Mediated Drug–Drug Interactions Using Population Pharmacokinetic Modeling and Simulation. Clin. Pharmacol. Ther. 2018, 104, 564–574. 10.1002/cpt.983. 29243231 PMC6175062

[ref34] Harrison J.; De Bruyn T.; Darwich A. S.; Houston J. B. Simultaneous Assessment In Vitro of Transporter and Metabolic Processes in Hepatic Drug Clearance: Use of a Media Loss Approach. Drug Metab. Dispos. 2018, 46, 405–414. 10.1124/dmd.117.079590. 29439129

[ref35] Ménochet K.; Kenworthy K. E.; Houston J. B.; Galetin A. Simultaneous Assessment of Uptake and Metabolism in Rat Hepatocytes: A Comprehensive Mechanistic Model. J. Pharmacol. Exp. Ther. 2012, 341, 2–15. 10.1124/jpet.111.187112. 22190645 PMC3310695

[ref36] Berry M. N.; Friend D. S. High-yield preparation of isolated rat liver parenchymal cells A Biochemical and Fine Structural Study. J. Cell Biol. 1969, 43, 506–520. 10.1083/jcb.43.3.506. 4900611 PMC2107801

[ref37] Mathworks. MATLAB R2017a; The MathWorks, Inc.: Natick, Massachusetts, United States, 2017.computer-program

[ref38] Davies B.; Morris T. Physiological Parameters in Laboratory Animals and Humans. Pharm. Res. 1993, 10, 1093–1095. 10.1023/a:1018943613122. 8378254

[ref39] Sourbron S. Technical Aspects of MR Perfusion. Eur. J. Radiol. 2010, 76, 304–313. 10.1016/j.ejrad.2010.02.017. 20363574

[ref40] Ziemian S.; Green C.; Sourbron S.; Jost G.; Schütz G.; Hines C. D. G. Ex Vivo Gadoxetate Relaxivities in Rat Liver Tissue and Blood at Five Magnetic Field Strengths from 1.41 to 7 T. NMR Biomed. 2021, 34, e4401 10.1002/nbm.4401. 32851735 PMC7757196

[ref41] Gertz M.; Tsamandouras N.; Säll C.; Houston J. B.; Galetin A. Reduced Physiologically-Based Pharmacokinetic Model of Repaglinide: Impact of OATP1B1 and CYP2C8 Genotype and Source of In Vitro Data on the Prediction of Drug-Drug Interaction Risk. Pharm. Res. 2014, 31, 2367–2382. 10.1007/s11095-014-1333-3. 24623479

[ref42] Tofts P. S.; Brix G.; Buckley D. L.; Evelhoch J. L.; Henderson E.; Knopp M. V.; Larsson H. B. W.; Lee T.-Y.; Mayr N. A.; Parker G. J. M.; Port R. E.; Taylor J.; Weisskoff R. M. Estimating Kinetic Parameters from Dynamic Contrast-Enhanced T1-Weighted MRI of a Diffusable Tracer: Standardized Quantities and Symbols. J. Med. Res. Inst. 1999, 10, 223–232. 10.1002/(sici)1522-2586(199909)10:3<223::aid-jmri2>3.0.co;2-s. 10508281

[ref43] Sourbron S. P.; Buckley D. L. On the Scope and Interpretation of the Tofts Models for DCE-MRI. Magn. Reson. Med. 2011, 66, 735–745. 10.1002/mrm.22861. 21384424

[ref44] Weinmann H.-J.; Schuhmann-Giampieri G.; Schmitt-Willich H.; Vogler H.; Frenzel T.; Gries H. A New Lipophilic Gadolinium Chelate as a Tissue-Specific Contrast Medium for MRI. Magn. Reson. Med. 1991, 22, 233–237. 10.1002/mrm.1910220214. 1812351

[ref45] Asaumi R.; Menzel K.; Lee W.; Nunoya K. i.; Imawaka H.; Kusuhara H.; Sugiyama Y. Expanded Physiologically-Based Pharmacokinetic Model of Rifampicin for Predicting Interactions With Drugs and an Endogenous Biomarker via Complex Mechanisms Including Organic Anion Transporting Polypeptide 1B Induction. CPT Pharmacometrics Syst. Pharmacol. 2019, 8, 845–857. 10.1002/psp4.12457. 31420941 PMC6875706

[ref46] Melillo N.; Grandoni S.; Cesari N.; Brogin G.; Puccini P.; Magni P. Inter-Compound and Intra-Compound Global Sensitivity Analysis of a Physiological Model for Pulmonary Absorption of Inhaled Compounds. AAPS J. 2020, 22, 116 10.1208/s12248-020-00499-0. 32862303 PMC7456635

[ref47] Hamby D. M. A Review of Techniques for Parameter Sensitivity Analysis of Environmental Models. Environ. Monit. Assess. 1994, 32, 135–154. 10.1007/bf00547132. 24214086

[ref48] Iooss B.; Lemaître P. A Review on Global Sensitivity Analysis Methods. Uncertainty Management in Simulation-Optimization of Complex Systems; Operations Research/Computer Science Interfaces Series; Springer: Boston, MA, 2015; pp 101–122.

[ref49] Archer G. E. B.; Saltelli A.; Sobol I. M. Sensitivity Measures, Anova-like Techniques and the Use of Bootstrap. J. Stat. Comput. Simulat. 1997, 58, 99–120. 10.1080/00949659708811825.

[ref50] Mathworks. MATLAB R2020a; The MathWorks, Inc.: Natick, Massachusetts, United States, 2020.computer-program

[ref51] Thai H.-T.; Mentré F.; Holford N. H. G.; Veyrat-Follet C.; Comets E. A Comparison of Bootstrap Approaches for Estimating Uncertainty of Parameters in Linear Mixed-Effects Models. Pharmaceut. Stat. 2013, 12, 129–140. 10.1002/pst.1561. 23457061

[ref52] Saltelli A.; Ratto M.; Andres T.; Campolongo F.; Cariboni J.; Gatelli D.; Saisana M.; Tarantola S. Global Sensitivity Analysis. The Primer; John Wiley & Sons, Ltd, 2008.

[ref53] Li R.; Maurer T. S.; Sweeney K.; Barton H. A. Does the Systemic Plasma Profile Inform the Liver Profile? Analysis Using a Physiologically Based Pharmacokinetic Model and Individual Compounds. AAPS J. 2016, 18, 746–756. 10.1208/s12248-016-9895-0. 26951483 PMC5256613

[ref54] Tsamandouras N.; Rostami-Hodjegan A.; Aarons L. Combining the ‘Bottom up’ and ‘Top down’ Approaches in Pharmacokinetic Modelling: Fitting PBPK Models to Observed Clinical Data. Br. J. Clin. Pharmacol. 2015, 79, 48–55. 10.1111/bcp.12234. 24033787 PMC4294076

[ref55] Watanabe T.; Kusuhara H.; Maeda K.; Shitara Y.; Sugiyama Y. Physiologically Based Pharmacokinetic Modeling to Predict Transporter-Mediated Clearance and Distribution of Pravastatin in Humans. J. Pharmacol. Exp. Ther. 2009, 328, 652–662. 10.1124/jpet.108.146647. 19001154

[ref56] Zamek-Gliszczynski M. J.; Chu X.; Cook J. A.; Custodio J. M.; Galetin A.; Giacomini K. M.; Lee C. A.; Paine M. F.; Ray A. S.; Ware J. A.; Wittwer M. B.; Zhang L.; ITC Commentary on Metformin Clinical Drug-Drug Interaction Study Design That Enables an Efficacy- and Safety-Based Dose Adjustment Decision. Clin. Pharmacol. Ther. 2018, 104, 781–784. 10.1002/cpt.1082. 29761830

[ref57] Kenna J. G.; Waterton J. C.; Baudy A.; Galetin A.; Hines C. D. G.; Hockings P.; Patel M.; Scotcher D.; Sourbron S.; Ziemian S.; Schuetz G. Noninvasive Preclinical and Clinical Imaging of Liver Transporter Function Relevant to Drug-Induced Liver Injury. In Drug-Induced Liver Toxicity; Chen M., Will Y., Eds.; Methods in Pharmacology and Toxicology; Springer: New York, NY, 2018; pp 627–651.

[ref58] Zamek-Gliszczynski M. J.; Lee C. A.; Poirier A.; Bentz J.; Chu X.; Ellens H.; Ishikawa T.; Jamei M.; Kalvass J. C.; Nagar S.; Pang K. S.; Korzekwa K.; Swaan P. W.; Taub M. E.; Zhao P.; Galetin A. ITC Recommendations for Transporter Kinetic Parameter Estimation and Translational Modeling of Transport-Mediated PK and DDIs in Humans. Clin. Pharmacol. Ther. 2013, 94, 64–79. 10.1038/clpt.2013.45. 23588311 PMC3898877

[ref59] Ishida K.; Ullah M.; Tóth B.; Juhasz V.; Unadkat J. D. Successful Prediction of In Vivo Hepatobiliary Clearances and Hepatic Concentrations of Rosuvastatin Using Sandwich-Cultured Rat Hepatocytes, Transporter-Expressing Cell Lines, and Quantitative Proteomics. Drug Metab. Dispos. 2018, 46, 66–74. 10.1124/dmd.117.076539. 29084782

[ref60] U.S. Food and Drug Administration. Drug Development and Drug Interactions: Table of Substrates, Inhibitors and Inducers; FDA, 2020, https://www.fda.gov/drugs/drug-interactions-labeling/drug-development-and-drug-interactions-table-substrates-inhibitors-and-inducers (accessed Dec 20 2020).web

[ref61] Barnett S.; Ogungbenro K.; Ménochet K.; Shen H.; Humphreys W. G.; Galetin A. Comprehensive Evaluation of the Utility of 20 Endogenous Molecules as Biomarkers of OATP1B Inhibition Compared with Rosuvastatin and Coproporphyrin I. J. Pharmacol. Exp. Ther. 2019, 368, 125–135. 10.1124/jpet.118.253062. 30314992

[ref62] Ufuk A.; Kosa R. E.; Gao H.; Bi Y.-A.; Modi S.; Gates D.; Rodrigues A. D.; Tremaine L. M.; Varma M. V. S.; Houston J. B.; Galetin A. In Vitro–In Vivo Extrapolation of OATP1B-Mediated Drug–Drug Interactions in Cynomolgus Monkey. J. Pharmacol. Exp. Ther. 2018, 365, 688–699. 10.1124/jpet.118.247767. 29643253

[ref63] Mori D.; Kimoto E.; Rago B.; Kondo Y.; King-Ahmad A.; Ramanathan R.; Wood L. S.; Johnson J. G.; Le V. H.; Vourvahis M.; David Rodrigues A.; Muto C.; Furihata K.; Sugiyama Y.; Kusuhara H. Dose-Dependent Inhibition of OATP1B by Rifampicin in Healthy Volunteers: Comprehensive Evaluation of Candidate Biomarkers and OATP1B Probe Drugs. Clin. Pharmacol. Ther. 2020, 107, 1004–1013. 10.1002/cpt.1695. 31628668 PMC7158214

[ref64] Chu X.; Liao M.; Shen H.; Yoshida K.; Zur A. A.; Arya V.; Galetin A.; Giacomini K. M.; Hanna I.; Kusuhara H.; Lai Y.; Rodrigues D.; Sugiyama Y.; Zamek-Gliszczynski M. J.; Zhang L. Clinical Probes and Endogenous Biomarkers as Substrates for Transporter Drug-Drug Interaction Evaluation: Perspectives From the International Transporter Consortium. Clin. Pharmacol. Ther. 2018, 104, 836–864. 10.1002/cpt.1216. 30347454

[ref65] Saito S.; Obata A.; Kashiwagi Y.; Abe K.; Murase K. Dynamic Contrast-Enhanced MRI of the Liver in Mrp2-Deficient Rats Using the Hepatobiliary Contrast Agent Gd-EOB-DTPA. Invest. Radiol. 2013, 48, 548–553. 10.1097/rli.0b013e3182856a06. 23442774

[ref66] Schabel M. C.; Parker D. L. Uncertainty and Bias in Contrast Concentration Measurements Using Spoiled Gradient Echo Pulse Sequences. Phys. Med. Biol. 2008, 53, 2345 10.1088/0031-9155/53/9/010. 18421121 PMC2894639

[ref67] Pfeifer N.; Goss S.; Swift B.; Ghibellini G.; Ivanovic M.; Heizer W.; Gangarosa L.; Brouwer K. Effect of Ritonavir on 99mTechnetium–Mebrofenin Disposition in Humans: A Semi-PBPK Modeling and In Vitro Approach to Predict Transporter-Mediated DDIs. CPT Pharmacometrics Syst. Pharmacol. 2013, 2, 20 10.1038/psp.2012.21. PMC3600725 23887590

