# Supplementary material for: Physiologically Based Pharmacokinetic Modeling of Transporter-Mediated Hepatic Disposition of Imaging Biomarker Gadoxetate in Rats
Source: Mol Pharm. 2021 Jul 20;18(8):2997–3009. doi: 10.1021/acs.molpharmaceut.1c00206 (PMC8397403; doi:10.1021/acs.molpharmaceut.1c00206)
Supplement: Supplementary file 1 — mp1c00206_si_001.pdf [file mp1c00206_si_001.pdf]

Physiologically-based pharmacokinetic modelling of transporter-mediated hepatic disposition of imaging biomarker gadoxetate in rats

Supplementary materials

Daniel Scotcher, Nicola Melillo, Sirisha Tadimalla, Adam S. Darwich, Sabina Ziemian, Kayode Ogungbenro, Gunnar Schütz, Steven Sourbron and Aleksandra Galetin*

*aleksandra.galetin@manchester.ac.uk

1. In vitro uptake of gadoxetate in plated rat hepatocytes

1.1. Uptake of gadoxetate and pitavastatin into plated rat hepatocytes

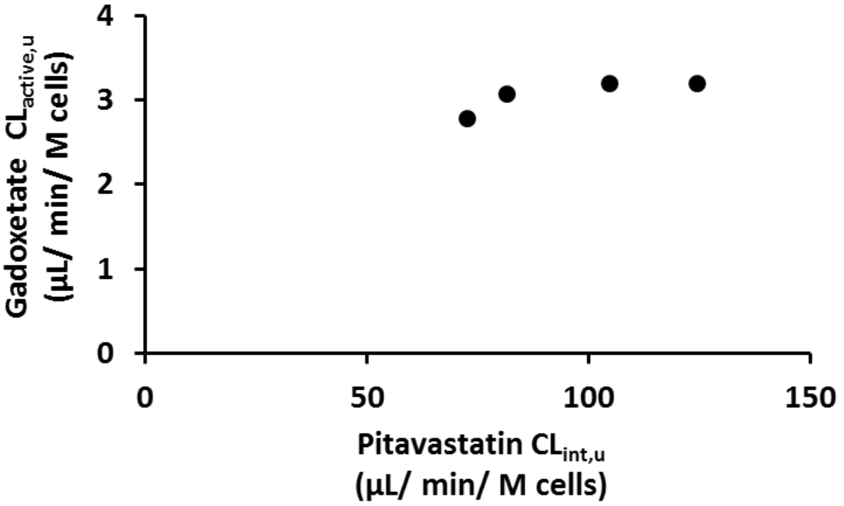

Figure S1: Uptake of gadoxetate and pitavastatin into plated rat hepatocytes

1.2. LC-MS/MS equipment and conditions

Table S1: LC-MS/MS equipment and conditions for quantification of gadoxetate and pitavastatin

	Gadoxetate	Pitavastatin
**Internal standard**	Diclofenac (0.1 µM)	Naloxone (0.1 µM)
**Liquid chromatography**		
**System**	Waters Alliance 2795 HPLC System [a]	Agilent 1100 HPLC system [b]
**Column**	Luna C18 3u 50x4.6mm [c]	Luna C18 3u 50x4.6mm [c]
**Flow rate (mL/min)**	1 [d]	1 [d]
**Injection volume (µL)**	20	10
**Solvents: A**	-	90% H_2O, 10% MeOH + 0.05% formic acid
**B**	-	10% H_2O, 90% MeOH + 0.05% formic acid
**C**	90% 5mM ammonium acetate at pH 5.6, 10% MeOH	90% H_2O, 10% MeOH + 1mM ammonium acetate
**D**	90% MeOH, 10% 5mM ammonium acetate at pH 5.6	10% H_2O, 90% MeOH + 1mM ammonium acetate

Gradient elution

Time (min)	A	B	C	D	Curve
**0**	-	-	90	10	1 [e]
**1**	-	-	90	10	1 [e]
**3**	-	-	10	90	6 [f]
**4**	-	-	10	90	1 [e]
**5**	-	-	90	10	1 [e]
**5.5**	-	-	90	10	1 [e]

Time (min)	A	B	C	D
**0**	-	-	100	-
**1**	-	-	100	-
**1.1**	50	50	-	-
**3**	-	100	-	-
**3.1**	-	-	-	100
**5**	-	-	-	100
**5.1**	-	-	100	-
**6.5**	-	-	100	-

**Analyte/ Internal standard retention time (min)**	2.8/ 4.0		4.2/ 3.0	
**Mass spectrometry**				
**System**	Waters Quattro Ultima Triple Quadrople Mass Spectrometer [a]		Micromass Quattro Ultima triple quadrupole mass spectrometer [a]	
**Ionisation**	Negative electrospray		Positive electrospray	
**Source/ desolvation temperature (°C)**	125/ 350		125/ 350	
**Desolvation/ cone gas flow rate (L/h)**	60/ 600		150/ 600	
**Capillary voltage (kV)**	2.65		3.5	
	Analyte	Internal standard	Analyte	Internal standard
**Cone voltage (V)**	110	30	95	50
**Collision voltage (eV)**	30	10	30	20
**Mass transition (m/z)**	680.9>635.05	293.95<249.95	422.9>291.0	328.85>310.75

[a] Waters, Elstree, Hertfordshire, UK.
[b] Aglient, Stockport, Cheshire, UK.
[c] Phenomenex, Macclesflield, UK.
[d] Split to deliver 0.25 mL/ min to mass spectrometer.
[e] Immediate switch.
[f] Linear transition.

2. PBPK model equations and residual calculation

The equations of the reduced PBPK model are reported in equation system (S1).

$$
\begin{aligned}
\frac{da_b}{dt} &= -\,Q_{CO}\frac{a_b}{V_b} - CL_r\frac{a_b}{V_b} + Q_{rob}\frac{a_{rob,v}}{V_{rob,v}\,K_{rob,v-b}} + Q_{liv}\frac{a_{liv,int}}{V_{liv,int}\,K_{liv,int-b}} \\
\frac{da_{rob,v}}{dt} &= Q_{rob}\left(\frac{a_b}{V_b} - \frac{a_{rob,v}}{V_{rob,v}}\right) - PS\left(\frac{a_{rob,v}}{V_{rob,v}} - \frac{a_{rob,ev}}{V_{rob,ev}}\right) \\
\frac{da_{rob,ev}}{dt} &= PS\left(\frac{a_{rob,v}}{V_{rob,v}} - \frac{a_{rob,ev}}{V_{rob,ev}}\right) \\
\frac{da_{spl,extr}}{dt} &= Q_{spl}\left(\frac{a_b}{V_b} - \frac{a_{spl,extr}}{V_{spl,extr}\,K_{spl-b}}\right) \\
\frac{da_{splan,extr}}{dt} &= Q_{splan}\left(\frac{a_b}{V_b} - \frac{a_{splan,extr}}{V_{splan,extr}\,K_{splan-b}}\right) \\
\frac{da_{liv,extr}}{dt} &= \left(Q_h - Q_{spl} - Q_{splan}\right)\frac{a_b}{V_b} + Q_{spl}\left(\frac{a_{spl,extr}}{V_{spl,extr}\,K_{spl-b}}\right) \\
&\quad + Q_{splan}\left(\frac{a_{splan,extr}}{V_{splan,extr}\,K_{splan-b}}\right) - Q_h\frac{a_{liv,extr}}{V_{liv,extr}\,K_{liv,extr-b}} \\
&\quad - CL_{active}\frac{a_{liv,extr}}{V_{liv,extr}} - CL_{passive}\left(\frac{a_{liv,extr}}{V_{liv,extr}} - f_{u,liv,cell}\frac{a_{liv,cell}}{V_{liv,cell}}\right) \\
\frac{da_{liv,cell}}{dt} &= CL_{active}\frac{a_{liv,extr}}{V_{liv,extr}} + CL_{passive}\left(\frac{a_{liv,extr}}{V_{liv,extr}} - f_{u,liv,cell}\frac{a_{liv,cell}}{V_{liv,cell}}\right) \\
&\quad - CL_{biliary}\,f_{u,liv,cell}\,\frac{a_{liv,cell}}{V_{liv,cell}}
\end{aligned}
\qquad \text{(S1)}
$$

a_τ, V_τ, Q_τ and $K_{\tau-b}$ represent the gadoxetate amount in the compartment τ, the compartment volume, blood flow and tissue to blood partition coefficient, respectively. Subscripts b, $spl, extr$ and $splan, extr$ are for blood and spleen and splanchnic organs extracellular space (considered to as blood in the organ plus interstitial space); rob, v and rob, ev are for vascular and extravascular (i.e., interstitial) rest of the body (ROB); $liv, extr$ and $liv, cell$ are for liver extracellular and liver intracellular (calculated as whole liver volume minus the extracellular liver volume). CL_r is the renal clearance, while PS is the permeability across the vascular endothelium of the ROB. CL_{active} and $CL_{passive}$ are the active and passive clearances across the hepatocytes cell membrane and $f_{u,liv,cell}$ is the gadoxetate fraction unbound in the hepatocytes intracellular compartment. $CL_{biliary}$ represent the elimination from the hepatocytes to the bile.

Gadoxetate does not distribute within the cells of other organs than the liver[1]. For this reason, we have considered just the extracellular volume of the spleen and splanchnic organs. Moreover, $V_{rob,ev}$ is the ROB interstitial space.

ROB is obtained following the lumping of lungs, brain, heart, kidneys, bones, thymus, muscles, skin and fat. Instead, the splanchnic compartment corresponds to the stomach, gut and pancreas. Volumes, blood flows and partition coefficients of the lumped compartments (τ_{lump}) were derived as follows (equation system (S2)).

$$\begin{aligned} V_{\tau_{lump}} &= \sum_i V_{\tau_i} \\ Q_{\tau_{lump}} &= \sum_i Q_{\tau_i} \\ K_{\tau_{lump}-b} &= \frac{\sum_i K_{\tau_i-b} \cdot V_{\tau_i}}{\sum_i V_{\tau_i}} \end{aligned} \tag{S2}$$

The vascular ROB to blood partition coefficient, $K_{rob,v-b}$, was considered equal to 1. The expression of the partition coefficients for spleen, splanchnic organs and liver is reported in equation (S3). As explained in section 3 of these supplementary materials, the expression in (S3) was derived with the hypothesis that plasma and interstitial gadoxetate concentration would reach equilibrium at the steady state.

$$K_{\tau-b} = \frac{V_{\tau,int} + V_{\tau,b} \cdot (1 - H_{ct})}{(V_{\tau,int} + V_{\tau,b}) \cdot (1 - H_{ct})} \tag{S3}$$

$V_{\tau,int}$ and $V_{\tau,b}$ are the interstitial and blood volume of the organ τ, while H_{ct} is the haematocrit.

3. Derivation of the partition coefficients

The objective here is to derive the tissue to blood partition coefficient, that can be expressed as

$$K_{\tau-b} = \frac{c_{\tau,extr}}{c_b}, \tag{S4}$$

where c_b and $c_{\tau,extr}$ are the gadoxetate concentration in blood and in the extracellular space of the tissue τ. The extracellular volume ($V_{\tau,extr}$) was supposed to be composed of the sum of the interstitial ($V_{\tau,int}$) and blood volume ($V_{\tau,b}$) in tissue τ.

$$V_{\tau,extr} = V_{\tau,int} + V_{\tau,b}$$

Gadoxetate was assumed to not distribute into the red blood cells[2]; it was considered to distribute into the residual plasma and in the interstitial space of the tissue τ, that are considered in instantaneous equilibrium. We can then define the volume of distribution of gadoxetate within a given tissue as follows.

$$V_{\tau,distr} = V_{\tau,int} + V_{\tau,b} \cdot (1 - H_{ct}),$$

Our main hypothesis is that gadoxetate plasma concentration (c_p) and its concentration in $V_{\tau,distr}$ ($c_{\tau,distr}$) will be in equilibrium at the steady state. Thus, we must find a $K_{\tau-b}$ that satisfies the following steady state relation.

$$c_p = c_{\tau,distr} \tag{S5}$$

It is possible to define a relation between c_p and c_b as follows, where V_p is the plasma volume.

$$c_p = \frac{a_b}{V_p} = \frac{a_b}{V_b(1 - H_{ct})} = \frac{c_b}{1 - H_{ct}}$$

Moreover, it is possible find a relation between $c_{\tau,extr}$ and $c_{\tau,distr}$ as well.

$$c_{\tau,extr} = \frac{a_{\tau,extr}}{V_{\tau,extr}} = \frac{a_{\tau,extr}}{V_{\tau,distr}} \cdot \frac{V_{\tau,distr}}{V_{\tau,extr}} = c_{\tau,distr} \frac{V_{\tau,distr}}{V_{\tau,extr}}$$

$$c_{\tau,distr} = c_{\tau,extr} \frac{V_{\tau,extr}}{V_{\tau,distr}}$$

Thus, by substituting the expressions of c_p and $c_{\tau,distr}$ in equation (S5), it is possible to derive the following equations.

$$c_{\tau,extr} \frac{V_{\tau,extr}}{V_{\tau,distr}} = \frac{c_b}{1 - H_{ct}}$$

Now, considering equation (S4), it is possible to derive the expression of $K_{\tau-b}$.

$$K_{\tau-b} = \frac{V_{\tau,distr}}{V_{\tau,extr} \cdot (1 - H_{ct})} = \frac{V_{\tau,int} + V_{\tau,b} \cdot (1 - H_{ct})}{\left(V_{\tau,int} + V_{\tau,b}\right) \cdot (1 - H_{ct})}$$

4. Calculation of the residuals

One difference between the DCE-MRI data used in this study with respect to the xenobiotic concentration commonly exploited within PBPK modelling, is that ΔR_1 values do not correspond uniquely to a specific time point. In fact, each data are acquired during a time interval Δt, that in the data used within the current PBPK analysis was equal to 57 *s.* To account for this characteristic, when performing the parameters estimation, the residuals were calculated as the difference of the observed ΔR_1 corresponding to a given time interval minus the mean of the simulated PBPK ΔR_1 within the same interval.

Let us consider $\Delta R_{1,data}(i)$ as the measured ΔR_1 for a given subject corresponding to the i-th time interval $\mathcal{T}_i = [t_i, t_i + \Delta t), i = 1 \dots N$, where N is the number of measurements for each subject and $t_i = t_{i-1} + \Delta t$, except for t_1, that is the time at which the experiment begins (assumed to be equal to 0). The residuals for each $\mathcal{T}_i$, res_i, were calculated as per equation (S6).

$$res_i = \Delta R_{1,data}(i) - \frac{1}{n_i} \sum_{\mathcal{t} \in \mathcal{T}_i} \Delta R_{1,PBPK}(\mathcal{t}) \qquad \text{(S6)}$$

$\Delta R_{1,PBPK}(\mathcal{t})$ are the ΔR_1 simulated with the PBPK model that correspond to a time $\mathcal{t} \in \mathcal{T}_i$ and n_i is their number. To assure that for each $\mathcal{T}_i$ there was the same number of equally spaced simulated $\Delta R_{1,PBPK}(\mathcal{t})$, the outputs of the PBPK model simulations were linearly interpolated to a fine grid of equally spaced time points, with distance equal to 0.01 *min*. To assess the sensitivity of the optimization on the choice of the time step of the grid, the analysis was repeated with a time step equal to 0.1 *min* and to the minimum time step of the output of the ordinary differential equations solver. In both the situations, no difference on the results of the analysis was observed.

5. PBPK model parameters

In **Table S2** the physiological PBPK parameters are reported. In the PBPK analysis, the rat weight was considered to be equal to 250 g, while the haematocrit was fixed to 0.418[3].

Table S2: physiological PBPK parameters for an average rat of 250 g

Organ	Blood flow [L/h][a]	Mass [g][b,c]	Vascular fraction[d]	Interstitial fraction[d]	Density [kg/L][e]
lungs	6.62	1.25	0.262	0.188	1.0505
brain	0.13	1.44	0.037	0.004	1.0355
heart	0.34	0.84	0.262	0.1	1.03
kidneys	0.93	1.84	0.105	0.2	1.05
bone	0.81	15	0.041	0.1	1.4303
muscle	1.84	101	0.026	0.12	1.041
stomach	0.08	1.15	0.032	0.1	1.05
spleen	0.053	0.5	0.282	0.15	1.054
liver	0.14[f]	9.15	0.115	0.163	1.08
gut	0.85[g]	5.6	0.024	0.094	1.043
pancreas	0.03	0.8	0.18	0.12	1.045
skin	0.38	47.58	0.019	0.302	1.183
fat	0.46	15.63	0.01	0.135	0.916
blood	6.62	15.77			1

[a] All the blood flows were taken from Brown *et al.*[4], except for the stomach, spleen and pancreas that were taken from Kawai et *al.*[5].
[b] Mass values are taken from Brown *et al.*[4], except for the blood, where the value for a rat of 250 g was derived from Lee and Blaufox[6].
[c] The organs volumes are derived from the mass divided for the density.
[d] All the fractions were taken from Kawai *et al.*[5].
[e] All the densities values were taken from Brown *et al.*[4], while the density of the blood was supposed equal to 1 kg/L.
[f] refers to the liver arterial blood flow.
[g] the gut blood flow was calculated as the liver portal blood flow from Brown *et al.*[4] minus the spleen, pancreas and stomach blood flows.

6. PBPK results

6.1. Bottom up PBPK approach

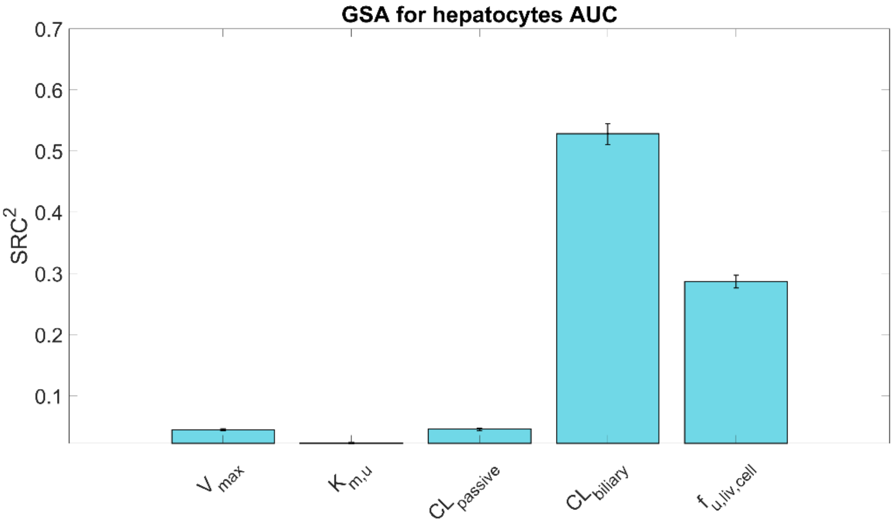

Figure S2: GSA results for the bottom up PBPK prediction, with the hepatocytes AUC considered as model output. The squared standardized regression coefficients (SRC^2) are reported in the barplot. Errorbars represent the 95% confidence interval of the estimated sensitivity indices, assessed with 1000 bootstrap samples. The higher the SRC^2 of a given parameter is, the more important that parameter is in explaining the hepatocytes AUC variance.

6.2. Top down PBPK approach

Here the bootstrap results (**Figure S3**) of the parameters identification and the simulation of the gadoxetate concentration in all the PBPK compartments are reported.

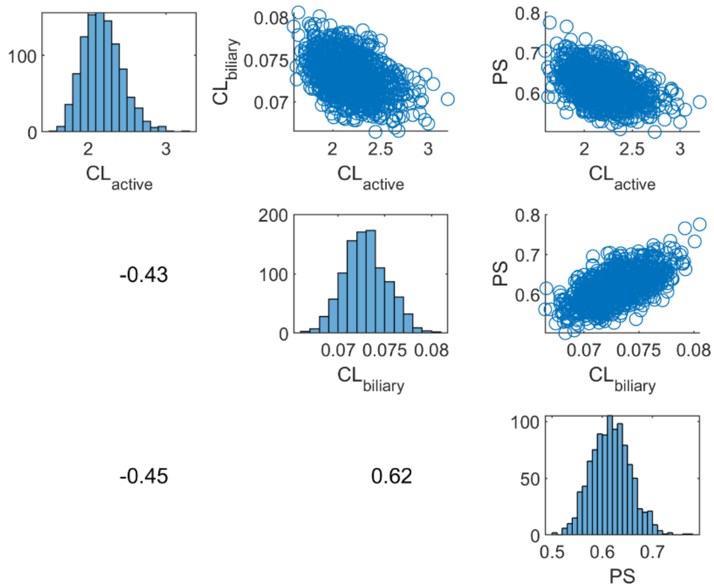

Figure S3: Bootstrap results of the top down approach. The case-bootstrap with 1000 samples was used to evaluate the uncertainty in the parameters estimates. In the diagonal, the bootstrap distribution of the parameters is reported; in the upper triangular, the scatterplots are reported; in the low triangular, the Pearson correlation coefficient is reported.

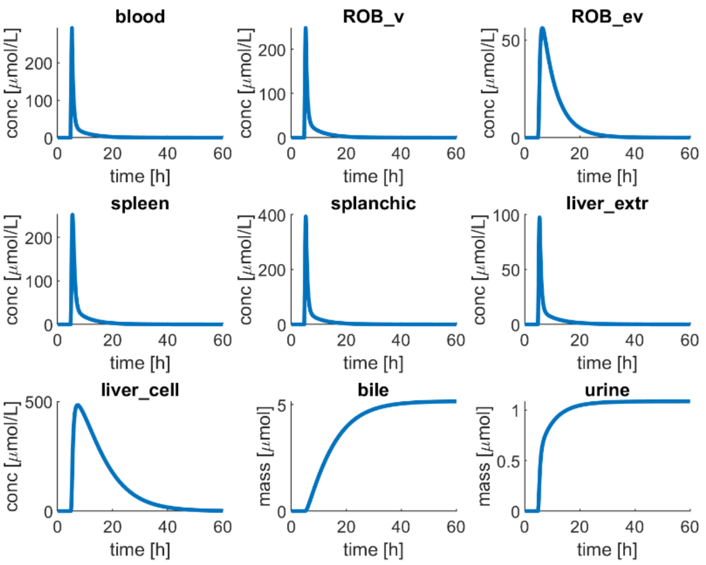

Figure S4: Plot of gadoxetate concentration-time curve in all the PBPK model compartments. 'ROB_v' and 'ROB_ev' stand for rest of the body vascular and extravascular (interstitial). 'liver_extr' and 'liver_cell' stand for liver extracellular and intracellular, respectively.

6.3. Top down PBPK approach – estimation with only the ΔR_1 blood data

Here we show the results of the parameters estimation performed only with the ΔR_1 blood data. As it is possible to see in **Table S3**, $CL_{biliary}$ was practically unidentifiable, while CL_{active} and PS are estimated with a relatively low coefficient of variation (CV). However, the liver profiles were not well captured (**Figure S5**).

Table S3: gadoxetate specific parameters in PBPK model estimated only with ΔR_1 blood data

Parameter name	Value[a]	Source
CL_{active} [L/h]	1.88 (23.2 %)	Estimated
$CL_{biliary}$ [L/h]	5 e-7 (>1000 %)	Estimated
PS [L/h]	1.92 (20.8 %)	Estimated
$CL_{passive}$ [L/h]	0.014	*In vitro* experiment[b]
$f_{u,liv,cell}$	0.648	*In vitro* experiment[b]
CL_r [L/h][c]	0.17	Literature

[a] for the estimated parameters, "Value" refers to the estimated value (CV), where CV is the coefficient of variation of the estimates, calculated with 1000 bootstrap samples.

[b] refers to the mean value in **Table 2** of the main manuscript.

[c] Calculated as CL_{total} x f_e, where CL_{total} is the total blood clearance, equal to 36.7 mL/min/kg and f_e is the fraction extracted in the urine, equal to 0.305[1].

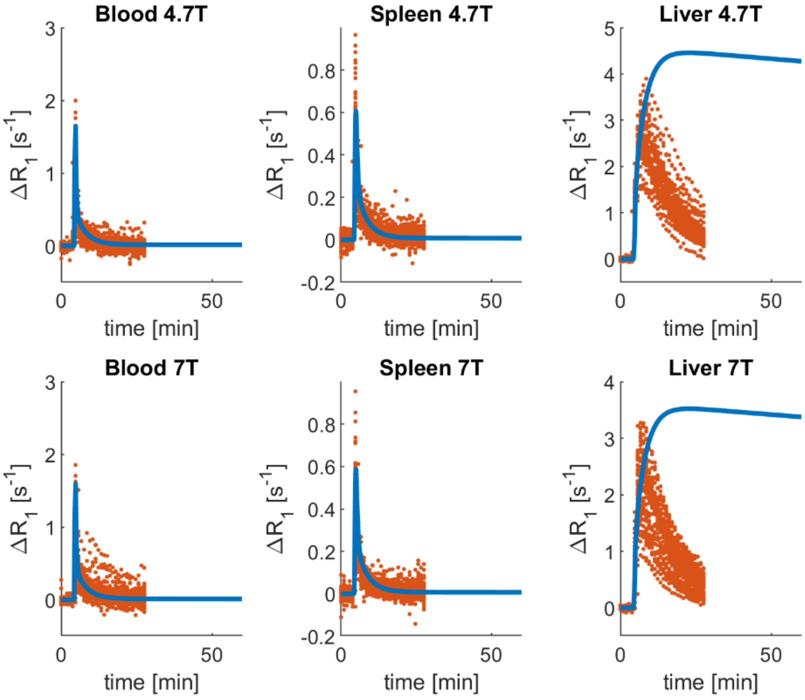

Figure S5: Results of fitting using the top down PBPK approach with the parameter identification performed only by using the blood ΔR_1 profiles. Red dots are the individual data points while the blue lines are the PBPK simulations. PBPK simulation and observed data versus time are reported for the blood, spleen and liver ΔR_1 at two field strengths, 4.7 T (first row) and 7 T (second row).

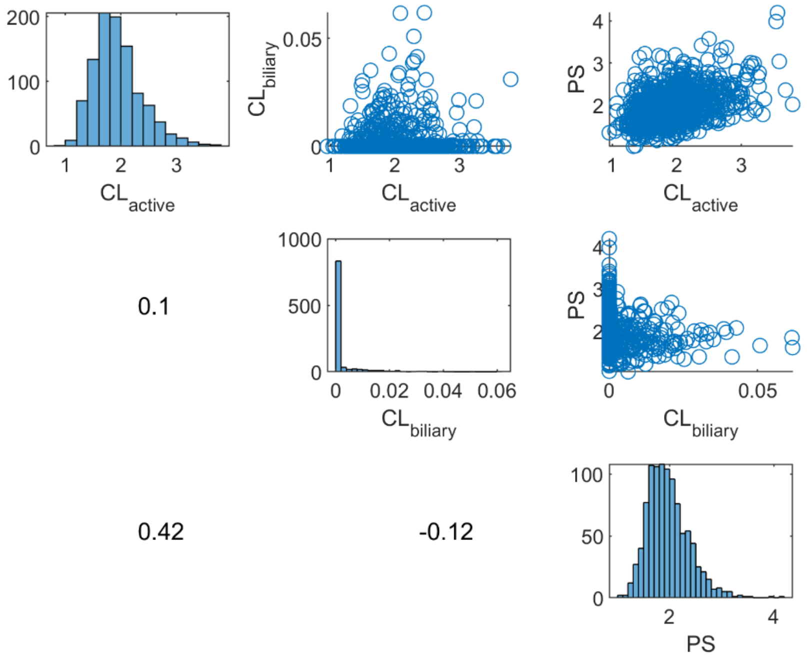

Figure S6: Results of the bootstrap of the top down PBPK approach with the parameters identification performed by using only the blood ΔR_1 profiles. The case-bootstrap with 1000 samples was used to evaluate the uncertainty in the parameter estimates. In the diagonal, the bootstrap distribution of the parameters is reported; in the upper triangular, the scatterplots are reported; in the low triangular, the Pearson correlation coefficient is reported.

6.4. Top down PBPK approach – estimation of CL_{active}, $CL_{biliary}$, CL_r and PS

Here we report the results of the simultaneous estimation of CL_{active}, $CL_{biliary}$, CL_r and PS. ΔR_1 profiles from the blood, spleen and liver data.

Table S4: Gadoxetate specific parameters following simultaneous estimation using deltaR1 data after administration of gadoxetate alone (absence of inhibitor)

Parameter name	Value[a]	Source
CL_{active} [L/h]	3.11 (>1000 %)	Estimated
$CL_{biliary}$ [L/h]	0.07 (2.6 %)	Estimated
PS [L/h]	0.58 (8.3 %)	Estimated
$CL_{passive}$ [L/h]	0.014	*In vitro* experiment[b]
$f_{u,liv,cell}$	0.648	*In vitro* experiment[b]
CL_r [L/h][c]	0.40 (24.6 %)	Estimated

[a] for the estimated parameters, "Value" refers to the estimated value (CV), where CV is the coefficient of variation of the estimates, calculated with 1000 bootstrap samples.
[b] refers to the mean value in **Table 2** of the main manuscript.
[c] Calculated as CL_{total} x f_e, where CL_{total} is the total blood clearance, equal to 36.7 mL/min/kg and f_e is the fraction extracted in the urine, equal to 0.305[1].

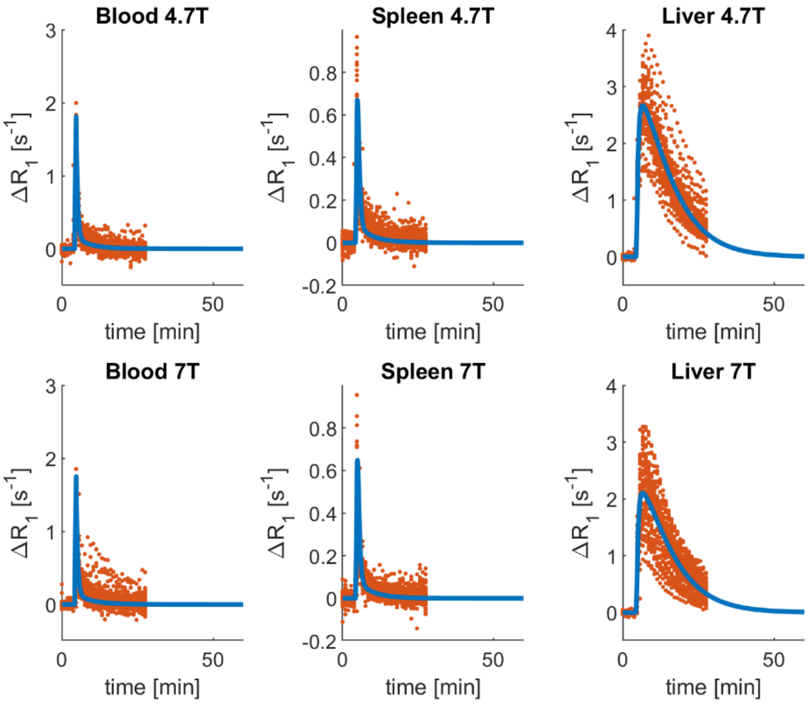

Figure S7: Results of fitting the top down PBPK approach with the simultaneous estimation of CL_r and PS. Red dots are the individual data points while the blue lines are the PBPK simulations. PBPK simulation and observed data versus time are reported for the blood, spleen and liver ΔR_1 at two field strengths, 4.7 T (first row) and 7 T (second row).

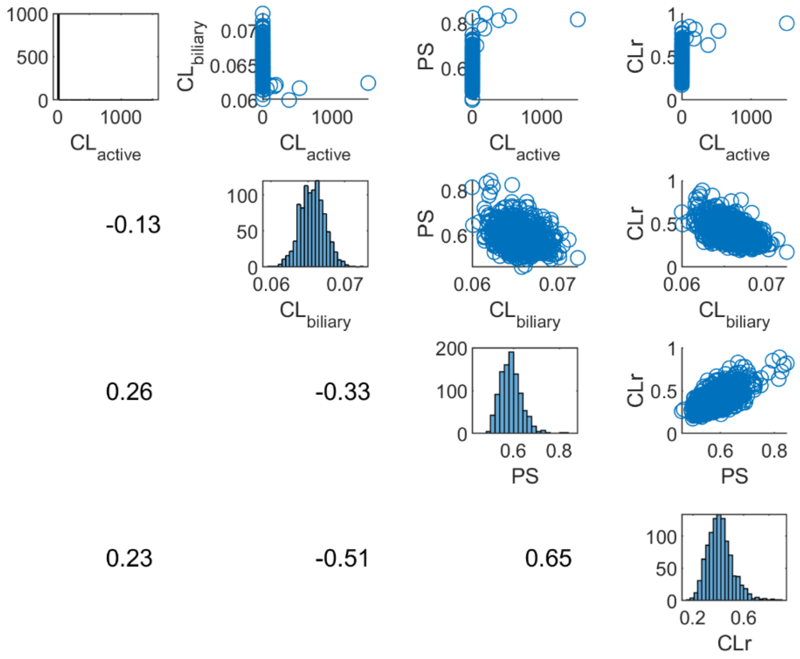

Figure S8: Results of bootstrap with the top down PBPK approach during simultaneous estimation of CL_r and PS. The case-bootstrap with 1000 samples was used to evaluate the uncertainty in the parameters estimates. In the diagonal, the bootstrap distribution of the parameters is reported; in the upper triangular, the scatterplots are reported; in the low triangular, the Pearson correlation coefficient is reported.

6.5. Simultaneous estimation of the control and inhibitory phase

Here the results of the simultaneous estimation of the control and inhibitory phases are reported.

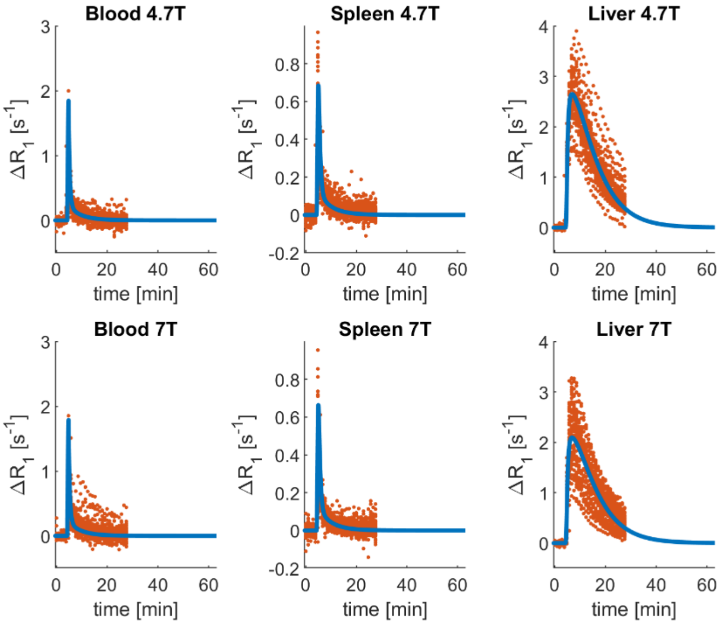

Figure S9: Results of fitting the PBPK model simultaneously to the control and inhibitory data. Red dots are the individual data points of the control phase of gadoxetate while the blue lines are the PBPK simulations. PBPK simulations and observed data are reported for the blood, spleen and liver ΔR_1 at two field strengths, 4.7 T (first row) and 7 T (second row).

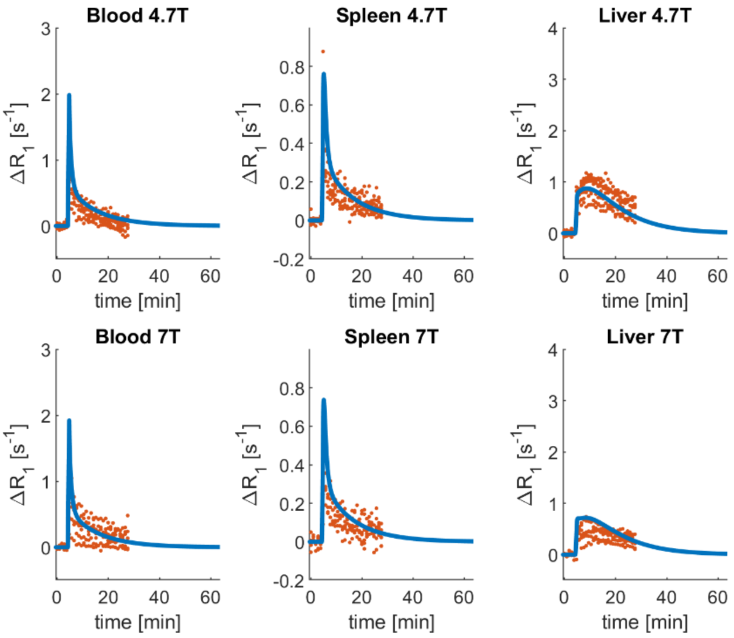

Figure S10: Results of fitting the PBPK model simultaneously to the control and inhibitory data. Red dots are the individual data points of gadoxetate administered with rifampicin while the blue lines are the PBPK simulations. PBPK simulation and observed data versus time profiles are reported for the blood, spleen and liver ΔR_1 at two field strengths, 4.7 T (first row) and 7 T (second row).

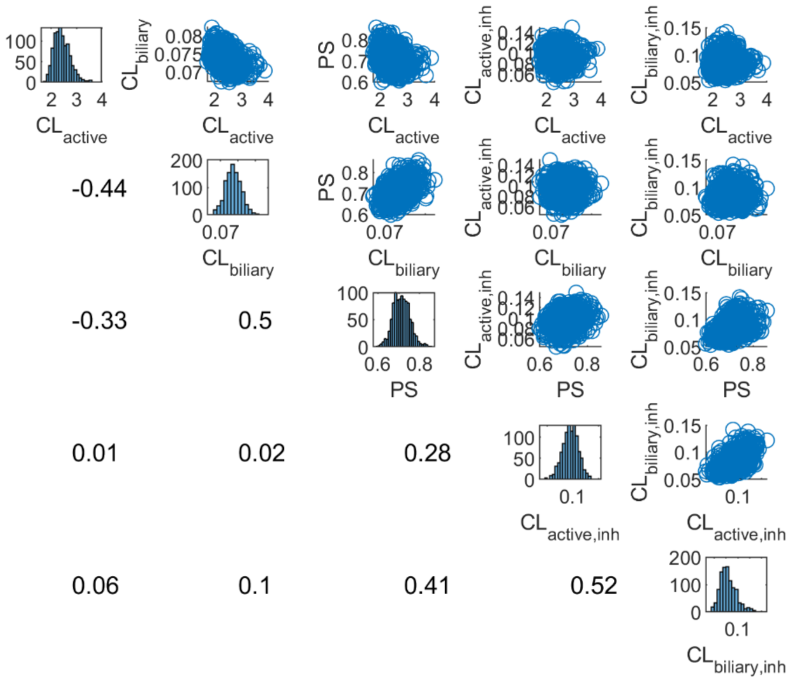

Figure S11: Results of the bootstrap from simultaneous estimation of parameters of the PBPK model using the control and inhibitory data. The case-bootstrap with 1000 samples was used to evaluate the uncertainty in the parameters estimates. In the diagonal, the bootstrap distribution of the parameters is reported; in the upper triangular, the scatterplots are reported; in the low triangular, the Pearson correlation coefficient is reported.

6.6. Simultaneous estimation of the control and inhibitory phase only with the blood ΔR_1 profiles

Table S5: Results of the parameter estimation from the simultaneously fitting the PBPK model to the control and inhibitory data by using only the blood ΔR_1

Parameter name	Value[a] control group	Value[a] rifampicin group
CL_{active} [L/h]	1.82 (30 %)	0.29 (45 %)
$CL_{biliary}$ [L/h]	3.3 e-5 (>1000 %)	1.3 e-8 (>1000%)
PS [L/h]	2.07 (20 %)	

[a] refers to the estimated value (CV), where CV is the coefficient of variation of the estimates, calculated with 1000 bootstrap samples.

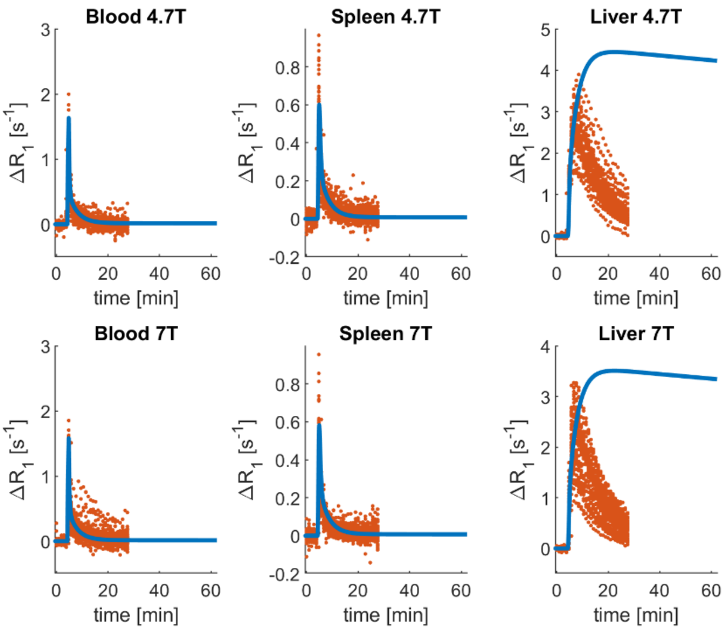

Figure S12: Results of fitting the PBPK model simultaneously to the control and inhibitory data using only the blood data. Red dots are the individual data points of gadoxetate administered alone while the blue lines are the PBPK simulations. PBPK simulations and observed data versus time profiles are reported for the blood, spleen and liver ΔR_1 at two field strengths, 4.7 T (first row) and 7 T (second row).

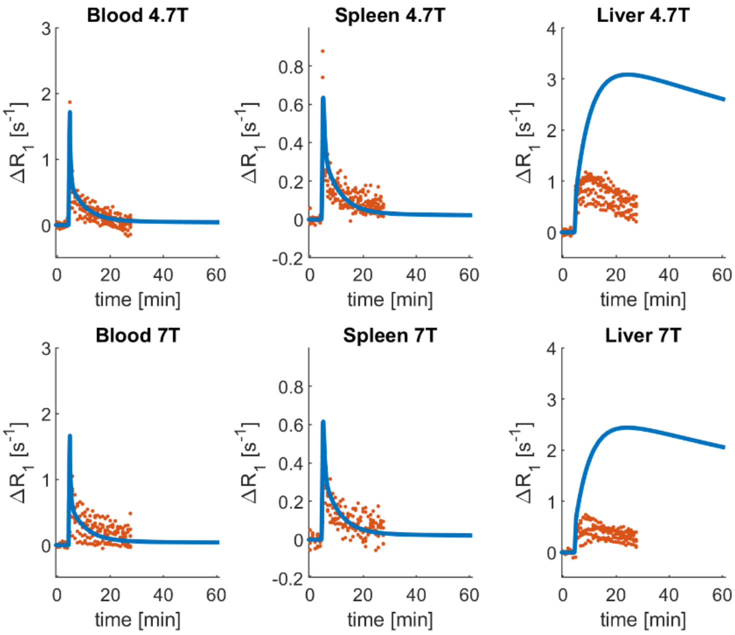

Figure S13: Results of fitting the PBPK model simultaneously to the data from control and inhibitory phases using only the blood data. Red dots are the individual data points of gadoxetate administered with rifampicin while the blue lines are the PBPK simulations. PBPK simulations and observed data versus time are reported for the blood, spleen and liver ΔR_1 at two field strengths, 4.7 T (first row) and 7 T (second row).

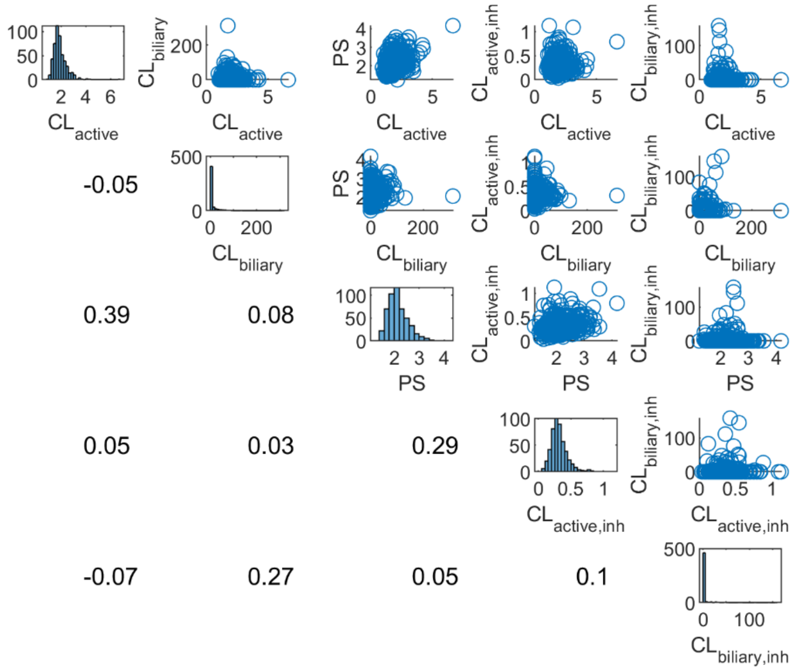

Figure S14: Results of the bootstrap from fitting the PBPK model simultaneously to the data from control and inhibitory phases using only the blood data. The case-bootstrap with 1000 samples was used to evaluate the uncertainty in the parameters estimates. In the diagonal, the bootstrap distribution of the parameters is reported; in the upper triangular, the scatterplots are reported; in the low triangular, the Pearson correlation coefficient is reported.

7. Impact of the number of liver compartments

Previous PBPK studies of hepatic transporter substrates have used a 5-compartment liver model[7]. Here our aim is to explore the impact of a 5-compartment liver model on the model prediction and parameters optimization. The equations used to describe the pharmacokinetics in the first compartments of both intracellular and extracellular liver are shown in equation system (S7), while the equations for the i-th compartment, with $i = 2, \dots, 5$, are reported in equation system (S8).

$$
\begin{aligned}
\frac{da_{liv,extr,1}}{dt} = {} & \left(Q_h - Q_{spl} - Q_{splan}\right)\frac{a_b}{V_b} + Q_{spl}\left(\frac{a_{spl,extr}}{V_{spl,extr}\,K_{spl-b}}\right) \\
& + Q_{splan}\left(\frac{a_{splan,extr}}{V_{splan,extr}\,K_{splan-b}}\right) - Q_h\frac{a_{liv,extr,1}}{V_{liv,extr}/n_{comp,liv}\,K_{liv,extr-b}} \\
& - CL_{active}/n_{comp,liv}\frac{a_{liv,extr,1}}{V_{liv,extr}/n_{comp,liv}} \\
& - CL_{passive}\left(\frac{a_{liv,extr,1}}{V_{liv,extr}/n_{comp,liv}} - f_{u,liv,cell}\frac{a_{liv,cell,1}}{V_{liv,cell}/n_{comp,liv}}\right) \\
\frac{da_{liv,cell,1}}{dt} = {} & CL_{active}/n_{comp,liv}\frac{a_{liv,extr,1}}{V_{liv,extr}/n_{comp,liv}} \\
& + CL_{passive}\left(\frac{a_{liv,extr,1}}{V_{liv,extr}/n_{comp,liv}} - f_{u,liv,cell}\frac{a_{liv,cell,1}}{V_{liv,cell}/n_{comp,liv}}\right) \\
& - CL_{biliary}/n_{comp,liv}\,f_{u,liv,cell}\frac{a_{liv,cell,1}}{V_{liv,cell}/n_{comp,liv}}
\end{aligned}
\tag{S7}
$$

$$
\begin{aligned}
\frac{da_{liv,extr,i}}{dt} = {} & +Q_h\frac{a_{liv,extr,i-1} - a_{liv,extr,i}}{V_{liv,extr}/n_{comp,liv}\,K_{liv,extr-b}} \\
& - CL_{active}/n_{comp,liv}\frac{a_{liv,extr,i}}{V_{liv,extr}/n_{comp,liv}} \\
& - CL_{passive}\left(\frac{a_{liv,extr,i}}{V_{liv,extr}/n_{comp,liv}} - f_{u,liv,cell}\frac{a_{liv,cell,i}}{V_{liv,cell}/n_{comp,liv}}\right) \\
\frac{da_{liv,cell,i}}{dt} = {} & CL_{active}/n_{comp,liv}\frac{a_{liv,extr,i}}{V_{liv,extr}/n_{comp,liv}} \\
& + CL_{passive}\left(\frac{a_{liv,extr,i}}{V_{liv,extr}/n_{comp,liv}} - f_{u,liv,cell}\frac{a_{liv,cell,i}}{V_{liv,cell}/n_{comp,liv}}\right) \\
& - CL_{biliary}/n_{comp,liv}\,f_{u,liv,cell}\frac{a_{liv,cell,i}}{V_{liv,cell}/n_{comp,liv}}
\end{aligned}
\tag{S8}
$$

where $a_{liv,extr,i}$ and $a_{liv,cell,i}$ are the i-th compartment of the extracellular and intracellular liver, respectively, $n_{comp,liv}$ is the number of liver compartments, here set equal to 5.

The results of the parameters identification following a simultaneous estimation using the control and inhibitory phases are reported in **Table S6**. The fitting results of the control and inhibitory phases are reported in **Figure S15** and **Figure S16**, respectively. The results of the case-bootstrap using 1000 samples are shown in **Figure S17**. It can be observed that comparing the results of the simultaneous estimation with the 5-compartment liver model (**Table S6**) with those of the 1-compartment liver model (**Table 4** in the main manuscript), the values of CL_{active} are one order of magnitude lower. However, even with the 5-compartment liver model a CL_{active} inhibition of 98% is estimated to occur due to the inhibition of rifampicin. Moreover, no significant change in the data description was observed.

Table S6: Results of the parameter estimation form a simultaneous fitting of the model to the data during control and inhibitory phases with the 5-compartment liver model

**Parameter name**	Value[a] control group	Value[a] rifampicin group
CL_{active} [L/h]	1.45 (7.4 %)	0.065 (28.2 %)
$CL_{biliary}$ [L/h]	0.07 (3.2 %)	0.07 (26.6 %)
PS [L/h]	0.81 (7.2 %)	

[a] refers to the estimated value (CV), where CV is the coefficient of variation of the estimates, calculated with 1000 bootstrap samples.

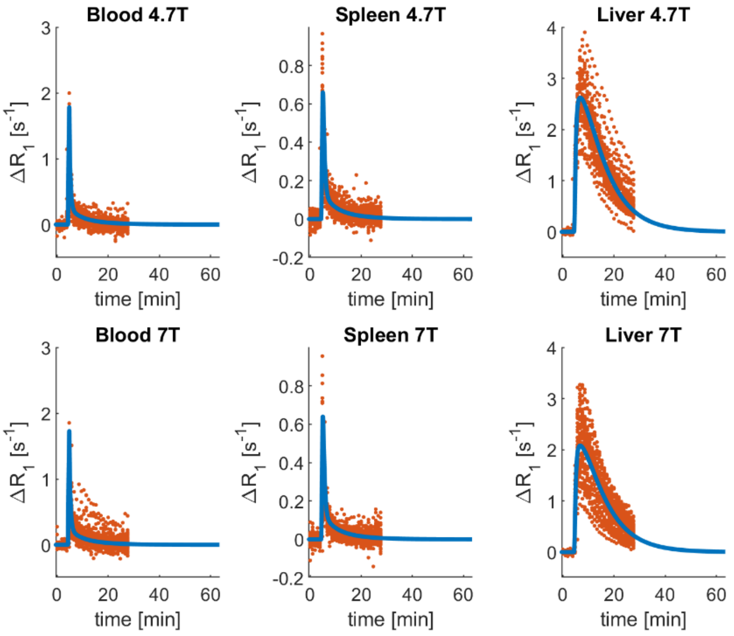

Figure S15: Results of the simultaneous estimation of parameters of the PBPK model from the data obtained from the control and inhibitory phases with the 5-compartment liver model. Red dots are the individual data

points of gadoxetate administered alone while the blue lines are the PBPK simulations. PBPK simulations and observed data versus time are reported for the blood, spleen and liver ΔR_1 at two field strengths, 4.7 T (first row) and 7 T (second row).

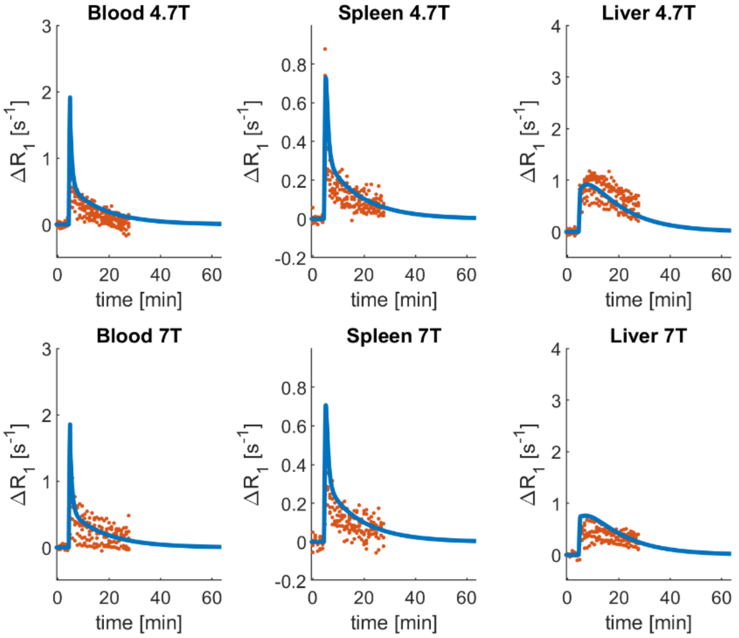

Figure S16: Results of the simultaneous fitting of the PBPK model to the data obtained during the control and inhibitory phases with the 5-compartment liver model. Red dots are the individual data points of gadoxetate administered together with rifampicin while the blue lines are the PBPK simulations. PBPK simulation and observed data versus time are reported for the blood, spleen and liver ΔR_1 at two field strengths, 4.7 T (first row) and 7 T (second row).

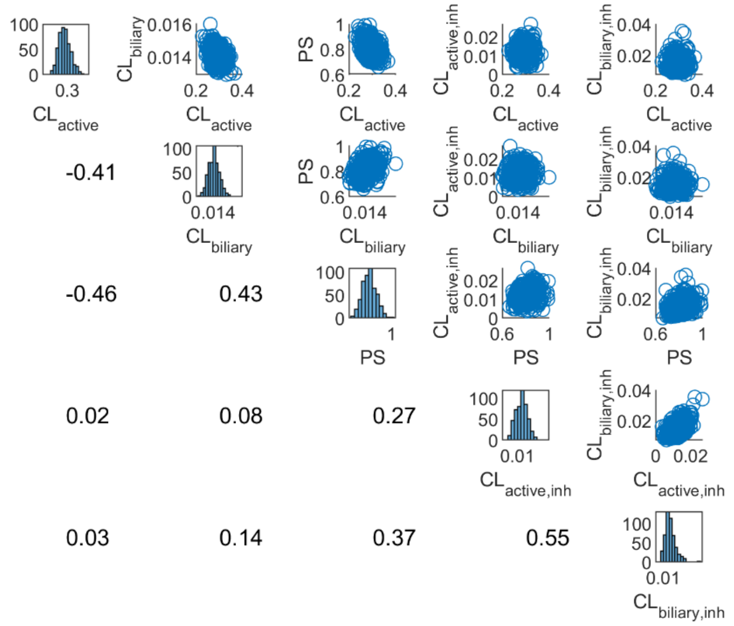

Figure S17: Results of the bootstrap from simultaneous estimation of the parameter of the PBPK model using the data obtained from the control and inhibitory phases using the 5-compartment liver model. The case-bootstrap with 1000 samples was used to evaluate the uncertainty in the parameters estimates. In the diagonal, the bootstrap distribution of the parameters is reported; in the upper triangular, the scatterplots are reported; in the low triangular, the Pearson correlation coefficient is reported.

References

1. Weinmann, H.-J. *et al.* A new lipophilic gadolinium chelate as a tissue-specific contrast medium for MRI. *Magn Reson Med* **22**, 233–237 (1991).
2. Forsgren, M. F., Leinhard, O. D., Dahlström, N., Cedersund, G. & Lundberg, P. Physiologically Realistic and Validated Mathematical Liver Model Revels Hepatobiliary Transfer Rates for Gd-EOB-DTPA Using Human DCE-MRI Data. *PLOS ONE* **9**, e95700 (2014).
3. Cremer, J. E. & Seville, M. P. Regional Brain Blood Flow, Blood Volume, and Haematocrit Values in the Adult Rat. *J Cereb Blood Flow Metab* **3**, 254–256 (1983).
4. Brown, R. P., Delp, M. D., Lindstedt, S. L., Rhomberg, L. R. & Beliles, R. P. Physiological Parameter Values for Physiologically Based Pharmacokinetic Models. *Toxicology and Industrial Health* **13**, 407–484 (1997).
5. Kawai, R., Mathew, D., Tanaka, C. & Rowland, M. Physiologically Based Pharmacokinetics of Cyclosporine A: Extension to Tissue Distribution Kinetics in Rats and Scale-up to Human. *Journal of Pharmacology and Experimental Therapeutics* **287**, 457–468 (1998).
6. Lee, H. B. & Blaufox, M. D. Blood Volume in the Rat. *J Nucl Med* **26**, 72–76 (1985).
7. Asaumi, R. *et al.* Expanded Physiologically-Based Pharmacokinetic Model of Rifampicin for Predicting Interactions With Drugs and an Endogenous Biomarker via Complex Mechanisms Including Organic Anion Transporting Polypeptide 1B Induction. *CPT:PSP* **8**, 845–857 (2019).
